# Supplementary material for: Comparative analysis of amplicon and metagenomic sequencing methods reveals key features in the evolution of animal metaorganisms
Source: Microbiome. 2019 Sep 14;7:133. doi: 10.1186/s40168-019-0743-1 (PMC6744666; doi:10.1186/s40168-019-0743-1)
Supplement: Supplementary file 1 — Supplementary Materials. (PDF 6900 kb) [file 40168_2019_743_MOESM1_ESM.pdf]

Comparative analysis of amplicon and metagenomic sequencing methods reveals key features in the evolution of animal metaorganisms

Philipp Rausch[1,2,3,†,*], Malte Rühlemann[4,†], Britt M. Hermes[1,2,5], Shauni Doms[1,2], Tal Dagan[6], Katja Dierking[7], Hanna Domin[8], Sebastian Fraune[8], Jakob von Frieling[9], Ute Hentschel[10,11], Femke-Anouska Heinsen[4], Marc Höppner[4], Martin T. Jahn[10], Cornelia Jaspers[11,12], Kohar Annie B. Kissoyan[7], Daniela Langfeldt[6], Ateeqr Rehman[4], Thorsten B. H. Reusch[11,12], Thomas Roeder[9], Ruth A. Schmitz[6], Hinrich Schulenburg[7], Ryszard Soluch[6], Felix Sommer[4], Eva Stukenbrock[13,14], Nancy Weiland-Bräuer[6], Philip Rosenstiel[4], Andre Franke[4], Thomas Bosch[8], John F. Baines[1,2,*]

Affiliations:

[1] Evolutionary Genomics, Max Planck Institute for Evolutionary Biology, Plön, Germany

[2] Institute for Experimental Medicine, Kiel University, Kiel, Germany

[3] Laboratory of Genomics and Molecular Biomedicine, Department of Biology University of Copenhagen, Copenhagen Ø, Denmark

[4] Institute of Clinical Molecular Biology, Kiel University, Kiel, Germany

[5] Lübeck Institute of Experimental Dermatology, University of Lübeck, Lübeck, Germany

[6] Institute of General Microbiology, Kiel University, Kiel, Germany

[7] Department of Evolutionary Ecology and Genetics, Zoological Institute, Kiel University, Kiel, Germany

[8] Zoological Institute, Kiel University, Kiel, Germany

[9] Molecular Physiology, Zoological Institute, Kiel University, Kiel, Germany

[10] Marine Ecology, Research Unit Marine Symbioses, GEOMAR Helmholtz Centre for Ocean Research, Kiel, Germany

[11] Kiel University, Kiel, Germany

[12] Marine Ecology, GEOMAR Helmholtz Centre for Ocean Research, Kiel, Germany

[13] Environmental Genomics, Max Planck Institute for Evolutionary Biology, Plön, Germany

[14] Environmental Genomics, Botanical Institute, Kiel University, Kiel, Germany

† Authors contributed equally

* Corresponding authors: Philipp Rausch (philipp.rausch@bio.ku.dk), John F. Baines (baines@evolbio.mpg.de)

Supplementary Material:

MEGAN [1] uses the information from all reads generated in the sequencing. Kraken uses a user-specified library of genomes as a database, where the records consist of a *k*-mer and the lowest common ancestor (LCA) whose genomes contain the k-mer. By querying the database for each *k*-mer in a sequence, and then using the resulting set of LCA taxa an appropriate label for the sequence can be determined. This drastically speeds up the classification process, but requires absolute matches which sacrifices sensitivity. MEGAN first needs a preprocessing step where reads are compared against a database (usually the non-redundant NCBI GeneBank database) using a BLAST algorithm or another comparison tools to search the top hits for each short read (here DIAMOND [2]). MEGAN then uses the NCBI taxonomy of the matched database to classify the sequences using a LCA algorithm, where reads are assigned to taxa such that the taxonomical level of the assigned taxon reflects the level of conservation of the sequence. MEGAN has the advantage that it simultaneously performs a taxonomical and functional classification of the reads simultaneously due to the inherent information of the database hits.

Supplementary Figures:

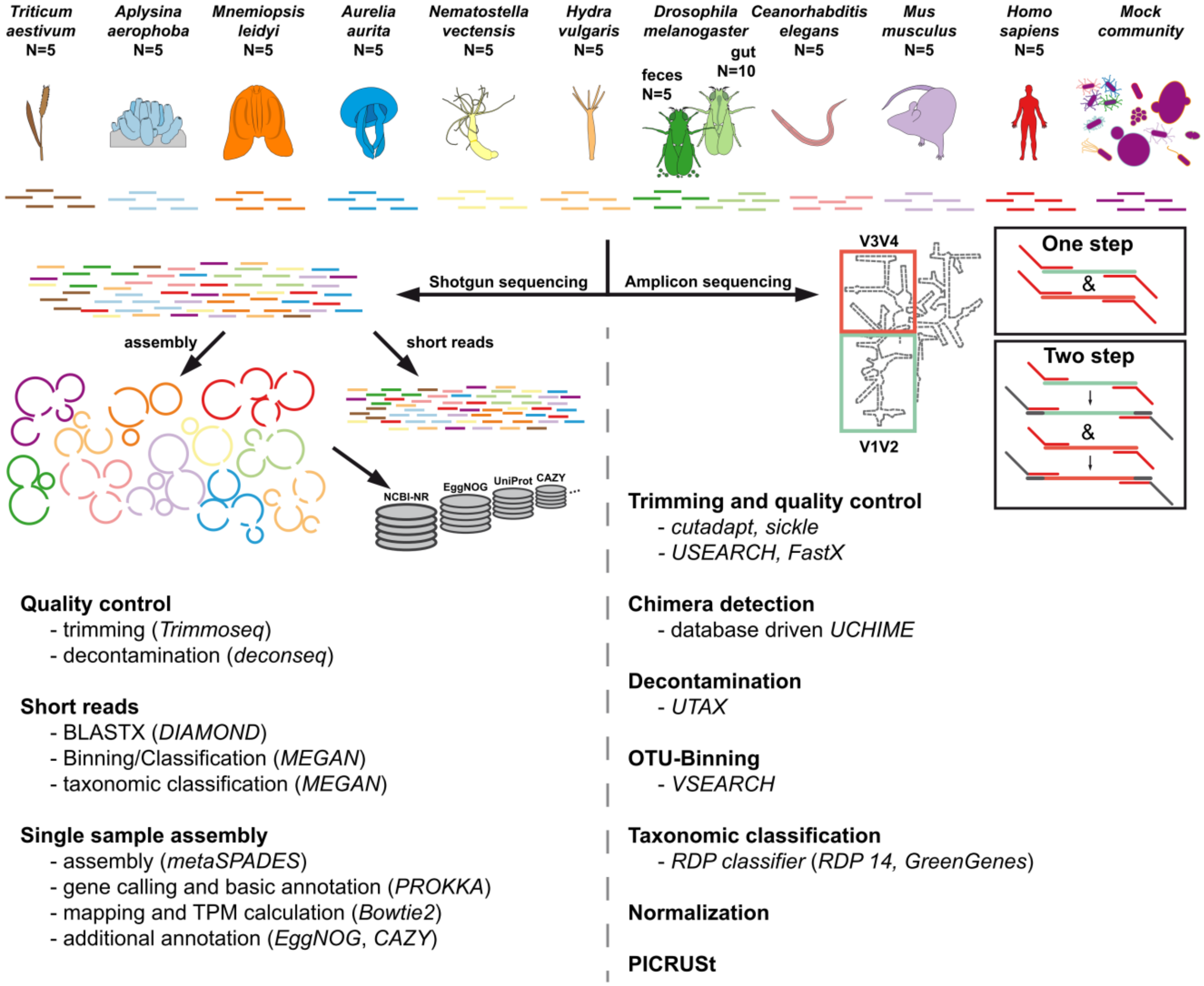

Figure S1: Visual abstract of the experiment, comparing different host organisms of the CRC 1182 investigated via different shotgun und 16S rRNA amplicon based methods (for final sample sizes see Table S1).

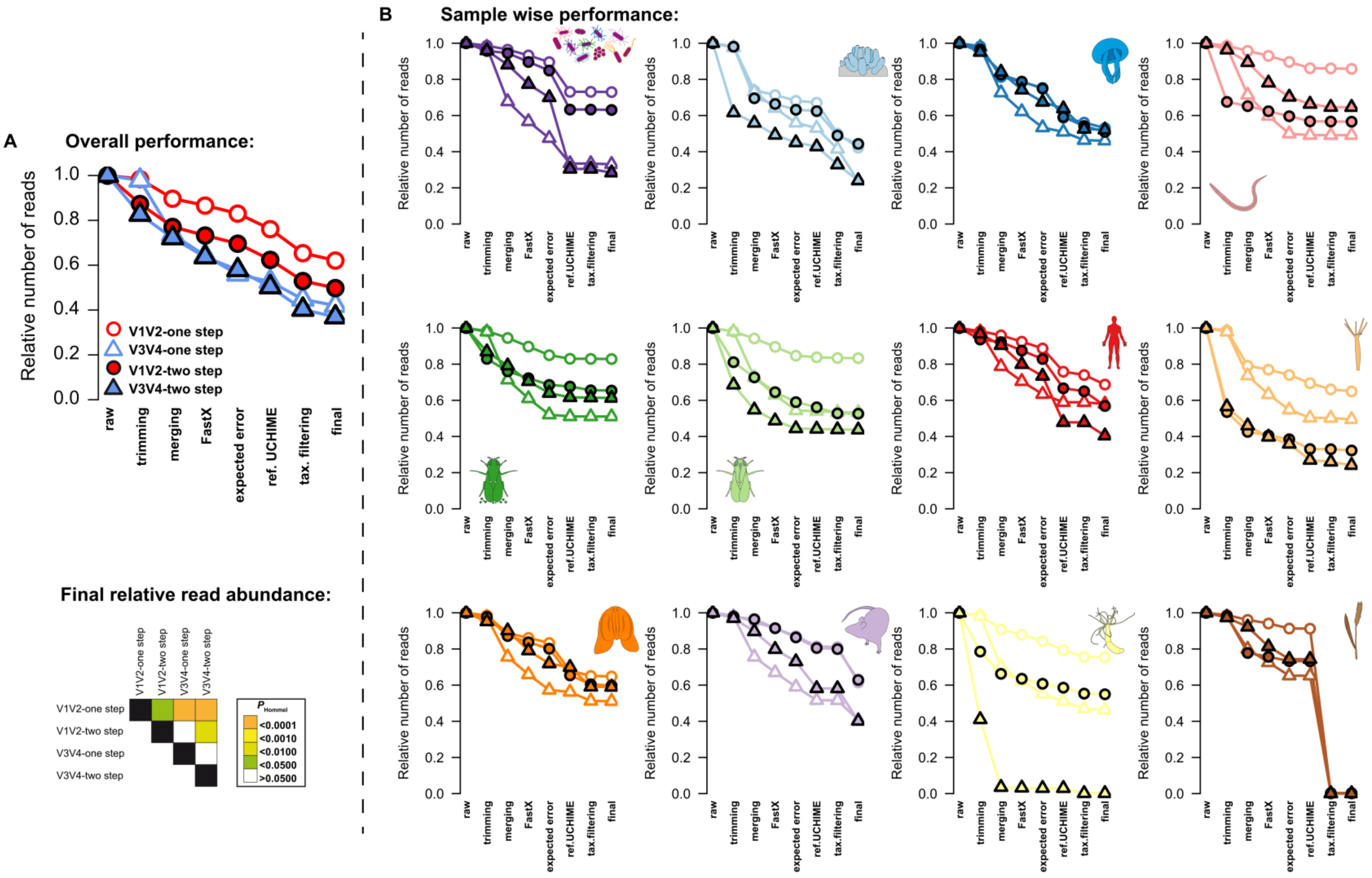

Figure S2: Average relative read count of the different 16S rRNA gene amplicon techniques (one step/two step, V1V2/V3V4) after each QC step employed in this study (for details please refer to the Methods section). (**A**) Average relative abundances of reads throughout the QC pipeline showing the significantly highest number of preserved reads in the V1V2-one step protocol (pairwise comparisons via pairwise *t*-Tests and Hommel *P*-value adjustment, significance levels are indicated by color). (**B**) Single plots display the average relative

read counts across the different QC steps for each sample/host type (mock community, *A. aerophoba*, *A. aurita*, *C. elegans*, *D. melanogaster* feces, *D. melanogaster* gut tissue, *H. sapiens*, *H. vulgaris*, *M. leidyi*, *M. musculus*, *N. vectensis*, *T. aestivum*).

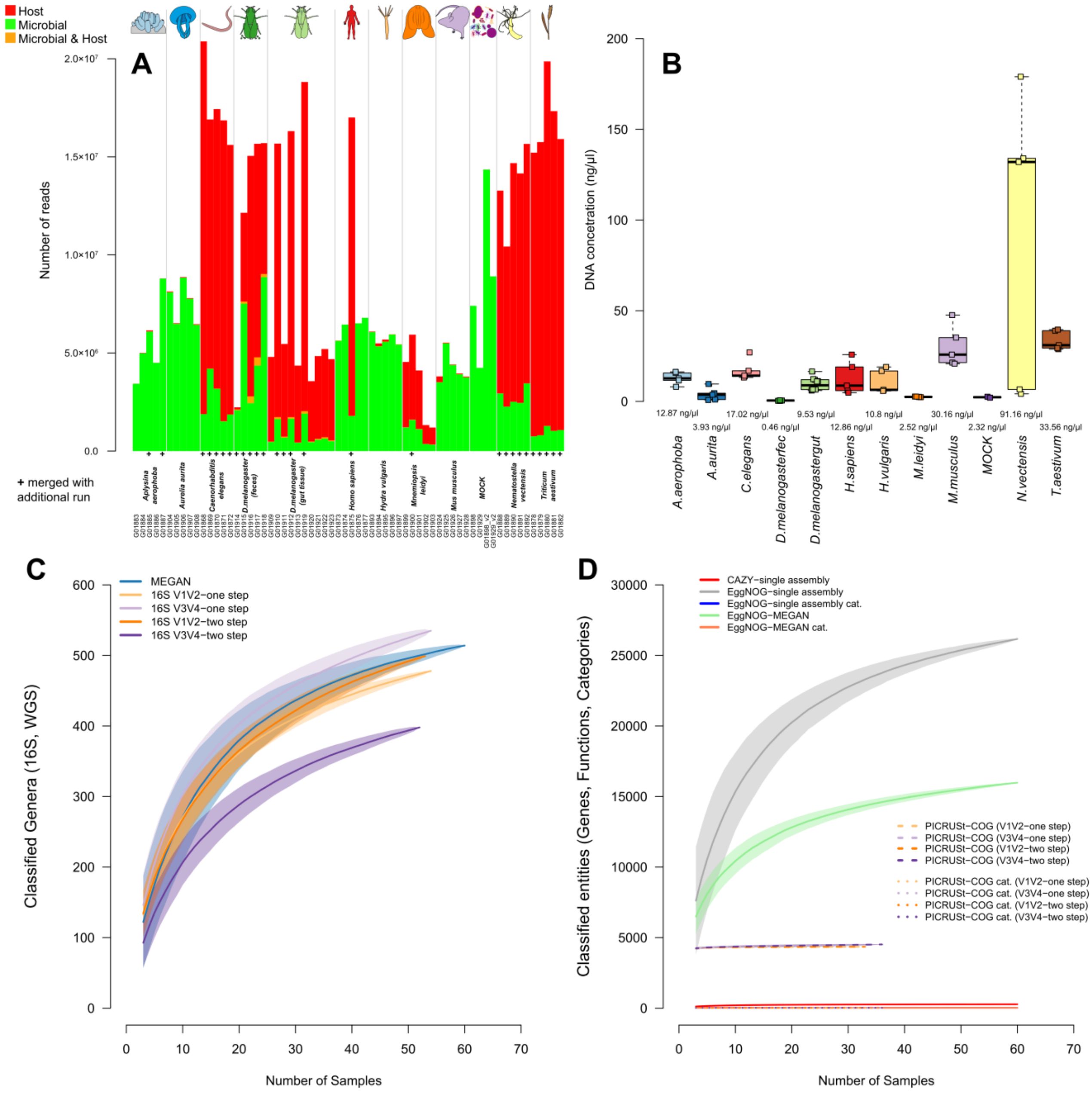

Figure S3: (A) Barplot displays the number of reads detected to be of host- or microbial origin for each sample. Resequenced samples are marked with a "+". **(B)** Average DNA concentration of samples (Qubit measurements). Collectors curves based on 1000 random re-samplings samples to see saturation of the number of **(C)** genera derived from shotgun and 16S rRNA gene amplicon techniques, as well as the number genes and functions **(D)** as derived from different shotgun based annotations and imputed functions (PICRUSt). Shading indicates standard deviations of the random samplings at each step (for sample sizes see Table S1).

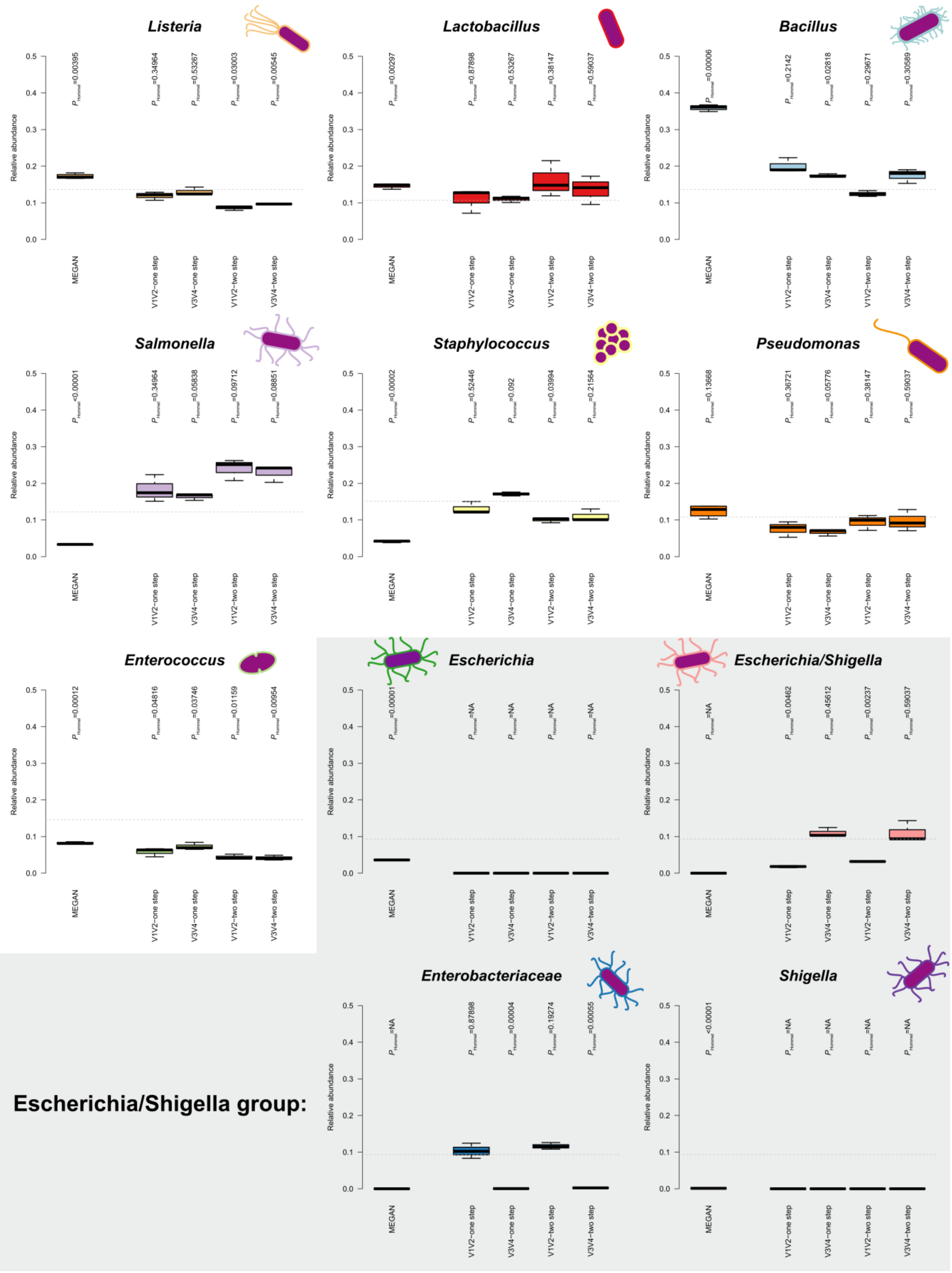
Listiera
Listeria
P_Hommel=0.00395
P_Hommel=0.34964
P_Hommel=0.53267
P_Hommel=0.03003
P_Hommel=0.00545
Lactobacillus
P_Hommel=0.00297
P_Hommel=0.87898
P_Hommel=0.53267
P_Hommel=0.38147
P_Hommel=0.59037
Bacillus
P_Hommel=0.00006
P_Hommel=0.2142
P_Hommel=0.02818
P_Hommel=0.29671
P_Hommel=0.30589
Salmonella
P_Hommel<0.00001
P_Hommel=0.34964
P_Hommel=0.05838
P_Hommel=0.09712
P_Hommel=0.08851
Staphylococcus
P_Hommel=0.00002
P_Hommel=0.52446
P_Hommel=0.092
P_Hommel=0.03994
P_Hommel=0.21564
Pseudomonas
P_Hommel=0.13668
P_Hommel=0.36721
P_Hommel=0.05776
P_Hommel=0.38147
P_Hommel=0.59037
Enterococcus
P_Hommel=0.00012
P_Hommel=0.04816
P_Hommel=0.03746
P_Hommel=0.01159
P_Hommel=0.00954
Escherichia
P_Hommel=0.00001
P_Hommel=NA
P_Hommel=NA
P_Hommel=NA
P_Hommel=NA
Escherichia/Shigella
P_Hommel=NA
P_Hommel=0.00462
P_Hommel=0.45612
P_Hommel=0.00237
P_Hommel=0.59037
Escherichia/Shigella group:
Enterobacteriaceae
P_Hommel=NA
P_Hommel=0.87898
P_Hommel=0.00004
P_Hommel=0.19274
P_Hommel=0.00055
Shigella
P_Hommel<0.00001
P_Hommel=NA
P_Hommel=NA
P_Hommel=NA
P_Hommel=NA
Relative abundance
0.0
0.1
0.2
0.3
0.4
0.5
MEGAN
V1V2−one step
V3V4−one step
V1V2−two step
V3V4−two step

Figure S4: Comparison of relative bacterial abundances in mock community samples to the expected relative abundances (dashed line) via one-sample Wilcoxon test (two-sided). Abundances are derived from 16S rRNA gene amplicon sequencing (V1V2, V3V4, one step, two step), MEGAN based classification (short reads). Sample sizes for the different approaches are $N_{shotgun}$=4, $N_{V1V2\text{-one step}}$=3, $N_{V1V2\text{-two step}}$=3, $N_{V3V4\text{-one step}}$=3, and $N_{V3V4\text{-two step}}$=3. *P*-values are corrected for multiple testing by Hommel *P*-value adjustment for each technique.

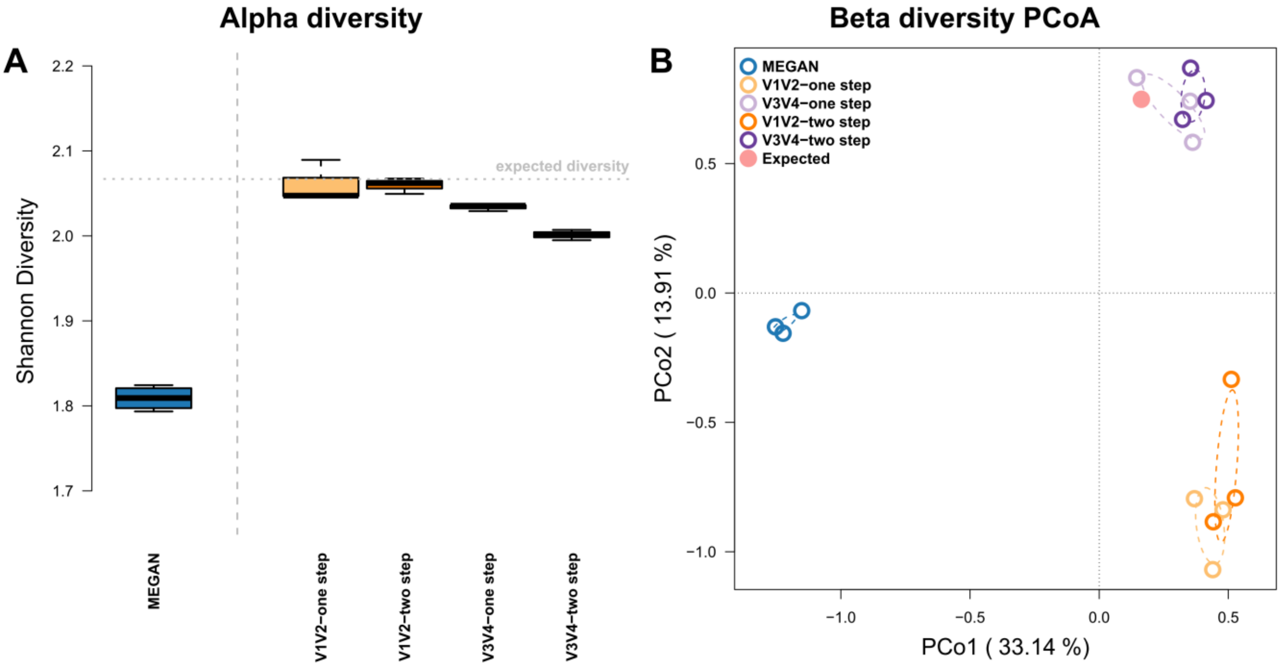

Figure S5: (A) Bacterial alpha diversity (Shannon H) derived via different techniques in comparison to the expected community diversity (dotted line). **(B)** Principle Coordinate Analyses (PCoA) of mock community compositions based on the different shotgun and 16S rRNA gene amplicon techniques focusing on shared occurrences (Jaccard). Ellipses represent standard deviations of points within the respective groups. Sample sizes for the different approaches are $N_{shotgun}$=4, $N_{V1V2\text{-one step}}$=3, $N_{V1V2\text{-two step}}$=3, $N_{V3V4\text{-one step}}$=3, and $N_{V3V4\text{-two step}}$=3.

Supplemental single host analyses:

Summary *Aplysina aerophoba* (Nardo, 1843):

Aplysina aerophoba is a Mediterranean-Atlantic member of the *Verongida*, which lives at light-exposed sites at depths from 5 to 15 m. *A. aerophoba*, as many demosponges, hosts a dense microbial community localized extracellularly in the mesohyl matrix which contributes to around one third of the sponge biomass [3, 4]. This sponge species serves as a model system to study basal host-microbial interactions and is further known for its biotechnological potential [5].

***Methods**:* Mediterranean *A. aerophoba* specimens were sampled offshore Girona, Spain, by scuba diving. Sponge specimens were rinsed with sterile sea water, fixed in RNAlater, and stored at -80°C. DNA was extracted from tissue via the Fast DNA Spin Kit for Soil (MP) with an additional ethanol precipitation step. No enrichment for bacterial DNA was used.

***Results**:* We see the highest alpha diversity in the shotgun profiles derived by MEGAN (Figure S6, left panel). The principle coordinate analysis of the beta diversities show a clear separation of shotgun vs amplicon-sequenced samples based on abundance and co-occurrence of genera (Figure S6, middle panel) and displays clustering of MEGAN based community profiles. Among the genera profiles derived from the 16S rRNA gene, there is a clear separation by variable region sequenced (*i.e.* V1V2 or V3V4) but less by amplification method. However, some samples based on the V3V4-two step method are clustering closely with MEGAN derived profiles (Figure S6, middle panel; Table S4), which also display a noticeable higher community variation (Figure S6, right panel).

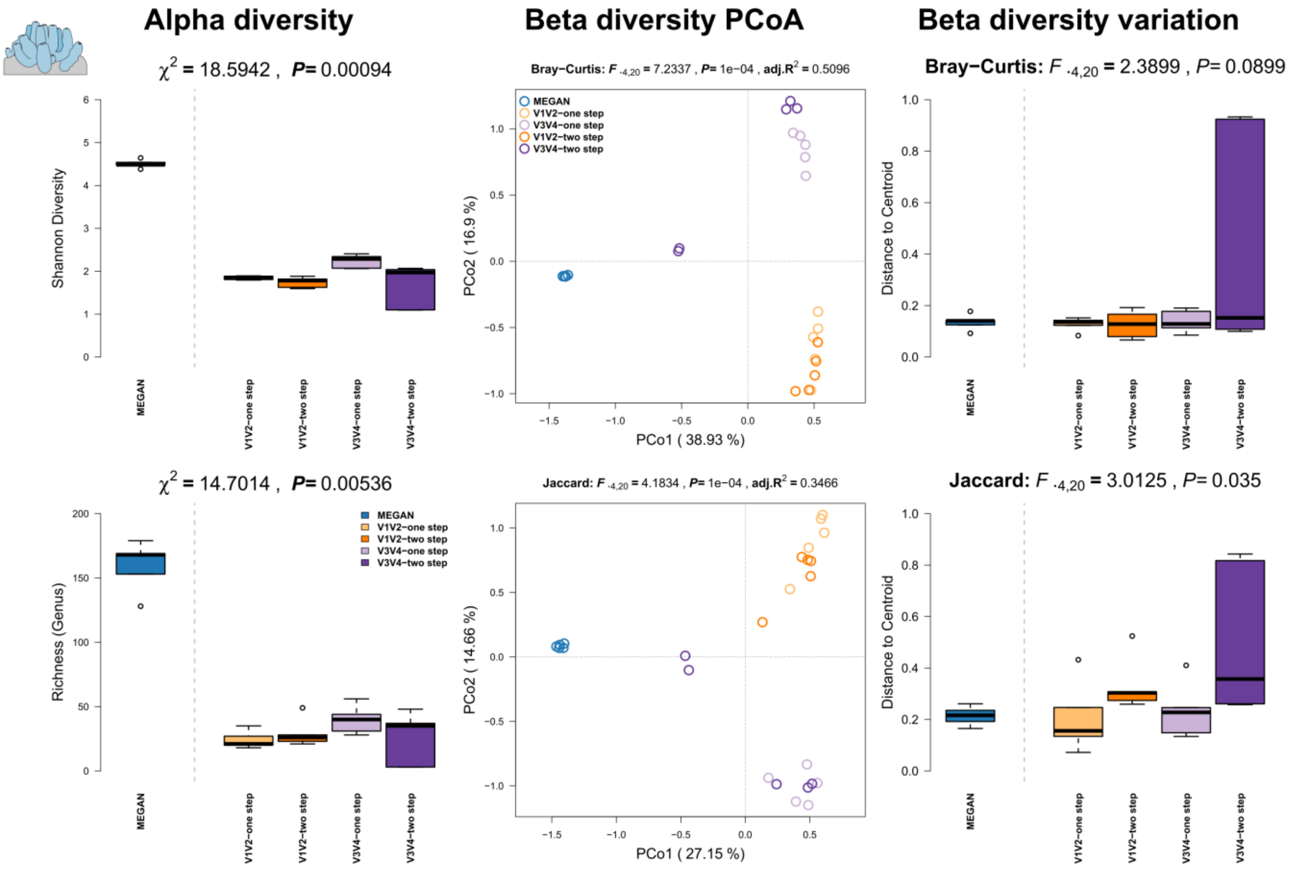

Figure S6: Comparison of community alpha diversity, composition, and variability of *Aplysina aerophoba* associated microbial communities among 16S rRNA gene amplicon and shotgun based pipelines. Comparisons are tested via approximate Kruskal-Wallis test [6] (alpha diversity), PERMANOVA [7, 8] (beta diversity), and permutative anova (variation of beta diversity). Community profiles were derived from 16S rRNA gene amplicon sequencing (V1V2, V3V4, one step, two step) and MEGAN based classification (short reads). Sample sizes for the

different approaches are $N_{shotgun}$=5, $N_{V1V2\text{-one step}}$=5, $N_{V1V2\text{-two step}}$=5, $N_{V3V4\text{-one step}}$=5, and $N_{V3V4\text{-two step}}$=5.

Summary *Aurelia aurita* (Linnaeus, 1758):

The moon jelly (*Aurelia aurita*) is a widely distributed pelagic scyphozoan found in almost all warm and temperate waters of coastal zones worldwide and is recognized as a key player in marine ecosystems [9]. Due to its ability to tolerate a wide range of environmental conditions, especially temperature and salinity, and its highly diverse food spectrum, it successfully colonizes different environments and often causes jellyfish blooms around the world [10].

***Methods**:* Individual *A. aurita* medusae (mean umbrella diameter 23 cm, N=5) were sampled from one location in the Eckernförder Bight, Baltic Sea (54.462654 N, 9.842743 E) in June 2016 by using a dip net. The animals were transported immediately to the laboratory, washed thoroughly with sterile filtered artificial seawater (ASW) to remove non-associated microbes. Pieces (2 × 2 cm) of the umbrella were cut out with a sterile scalpel. Dissociation of tissues was performed overnight at 4°C with 1 mg/mL collagenase (Sigma-Aldrich, St. Louis/USA). Homogenates were filtered through 10 µm Nylon gaze, followed by adding 0.1% IGEPAL CA-630 (Sigma-Aldrich, St. Louis/USA) and centrifugation of samples for 25 min at 300 × g at 4 °C. Supernatant including the prokaryotic fraction was centrifuged 5 min at 7,500 × g. DNA was extracted using the Wizard genomic purification kit (Promega, Madison, WI, USA). Pellets from eukaryotic/prokaryotic cell separation were homogenized in 480 µl 50mM EDTA and incubated at 37°C for 30 min after the addition of 10 mg/mL lysozyme (Carl Roth, Karlsruhe/Germany) and 60 Units Proteinase K (Life Technologies, Darmstadt/Germany). The remaining preparation steps were performed according to the manufacturer's protocol.

***Results**:* Alpha diversity estimates are mostly comparable between shotgun- and 16S rRNA amplicon -based community profiles (Figure S7, left panel). MEGAN however, detects a lower number of genera compared to amplicon techniques. The 16S rRNA gene amplicon profiles are homogeneous and only show a slightly higher diversity in the V3V4 based community profiles. The principle coordinate analyses show a relatively close clustering of MEGAN and the V1V2 profiles which together are separated from the V3V4 profiles based on shared abundances of genera. However, there is a clear separation among the shotgun- and two amplicon-derived profiles when we only consider the presence of bacterial genera among samples (Figure S7, middle panel; Table S4). Within the 16S rRNA gene amplicon profiles, there is a separation by variable region based on abundance differences between samples (*i.e.* V1V2 or V3V4). When we focus on community variation we see a slightly higher variation in beta diversity in one-step PCR protocols compared to the two-step PCR protocols and MEGAN, although not significant (Figure S7, right panel).

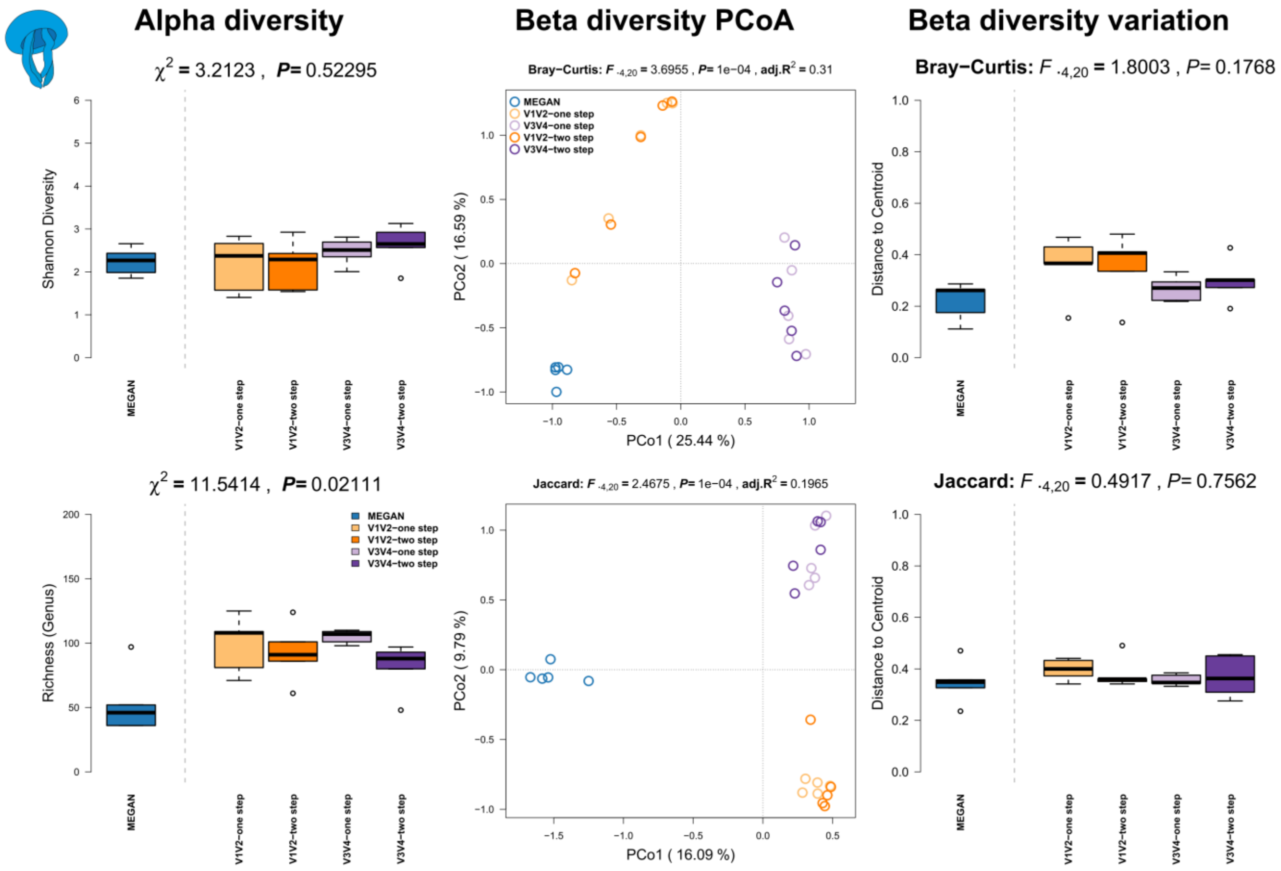

Figure S7: Comparison of community alpha diversity, composition, and variability of *Aurelia aurita* associated microbial communities among 16S rRNA gene amplicon and shotgun based pipelines. Comparisons are tested via approximate Kruskal-Wallis test (alpha diversity), PERMANOVA (beta diversity), and permutative anova (variation of beta diversity). Community profiles were derived from 16S rRNA gene amplicon sequencing (V1V2, V3V4, one step, two step), MEGAN based classification (short reads). Sample sizes for the different approaches are $N_{shotgun}$=5, $N_{V1V2\text{-one step}}$=5, $N_{V1V2\text{-two step}}$=5, $N_{V3V4\text{-one step}}$=5, and $N_{V3V4\text{-two step}}$=5.

Summary *Caenorhabditis elegans* (Maupas, 1900):

The nematode *Caenorhabditis elegans* provides a multitude of experimental advantages, such as small size, large-scale culturing, short generation time, transparency, genetic tractability, and thus, it has become one of the most widely used model organisms in biological research. In the laboratory *C. elegans* is almost exclusively cultivated mono-axenically on its food bacterium *E. coli* OP50. Therefore, almost all of the numerous studies with this nematode ignore a potential influence of the microbiome. In contrast, natural *C. elegans* are associated with a wide range of different microorganisms. Hence, wild caught and naturally colonized worms are of great interest to study the influence of a natural bacterial flora on nematode biology and fundamental developmental and genetic pathways.

***Methods**: C. elegans* were isolated directly from compost (n=2) and slugs found on the same compost (n=3) in the botanical garden in Kiel, Northern Germany (54°200N and 10°060E) in 2012, as previously described [11-13]. The samples were frozen in 30% glycerol-TSB and stored at -80°C. Worm samples were thawed and washed three times with M9 buffer with 0.05% Triton X-100 prior to DNA isolation [14]. DNA extractions from worm samples and a negative control (nuclease free water) were performed using the CTAB (Cetyl Trimethyl Ammonium Bromide) protocol as previously described [11, 13, 15]. No enrichment procedure for bacterial DNA was used.

***Results**:* We can observe no differences in alpha diversity between the shotgun- and 16S rRNA amplicon based community profiles (Figure S8, left panel). Communities further do not differ significantly between methods according to the bacterial abundances, but do slightly between MEGAN and 16S rRNA based techniques when we only consider the shared presence of genera (Figure S8, middle panel; Table S4). Furthermore, the different techniques do not differ in the amount of community variation.

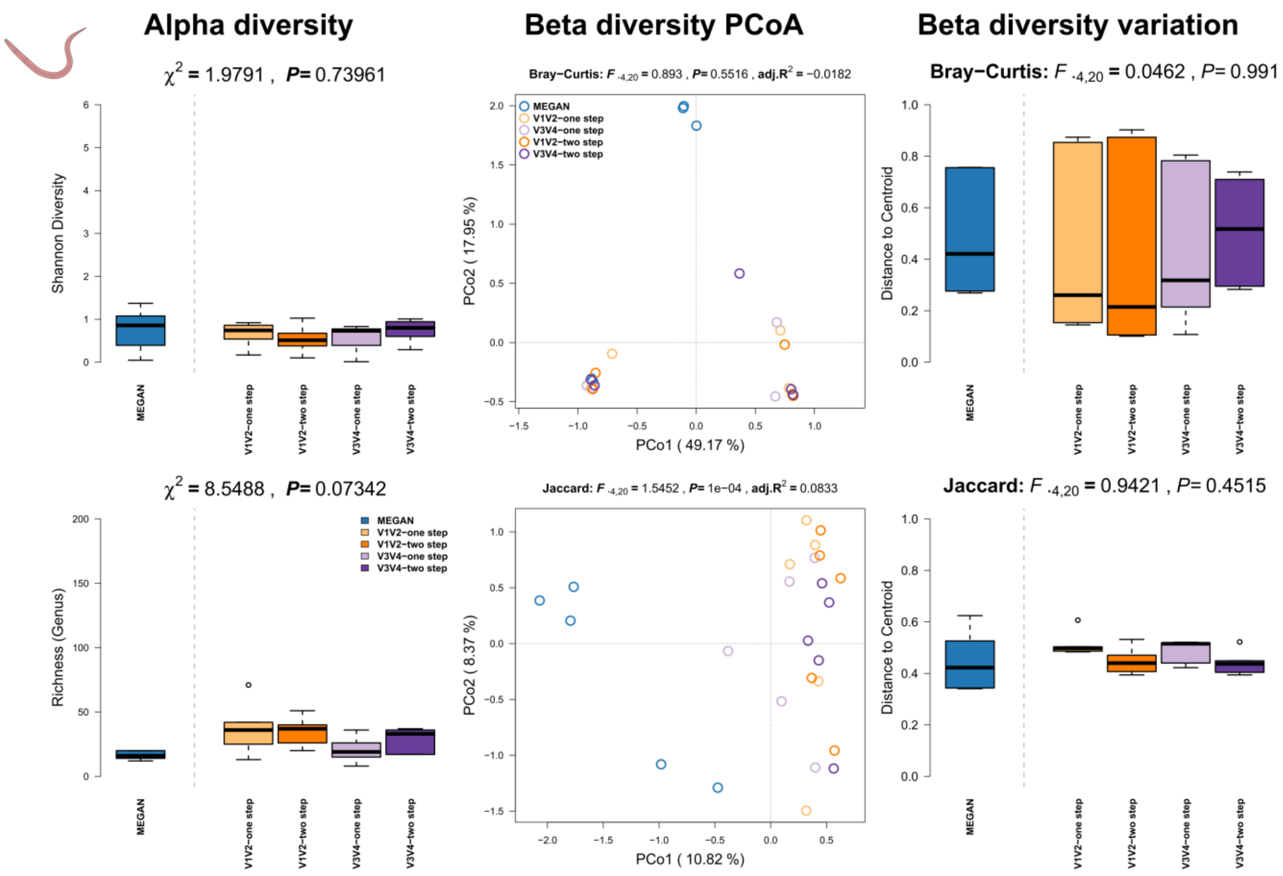

Figure S8: Comparison of community alpha diversity, composition, and variability of *Caenorhabditis elegans* associated microbial communities among 16S rRNA gene amplicon and shotgun based pipelines. Comparisons are tested via approximate Kruskal-Wallis test (alpha diversity), PERMANOVA (beta diversity), and permutative anova (variation of beta diversity). Community profiles were derived from 16S rRNA gene amplicon sequencing (V1V2, V3V4, one

step, two step), MEGAN based classification (short reads). Sample sizes for the different approaches are $N_{shotgun}$=5, $N_{V1V2\text{-one step}}$=5, $N_{V1V2\text{-two step}}$=5, $N_{V3V4\text{-one step}}$=5, and $N_{V3V4\text{-two step}}$=5.

Summary *Drosophila melanogaster* (Meigen, 1830):

Drosophila melanogaster is one of the best established model organisms for genetic and developmental investigations with a plethora of tools and protocols available for genetic, physiological and neurological manipulations and was the first metazoan genome to be sequenced [16]. Due to its rather simple metagenome, its easy husbandry, and well established tools, *D. melanogaster* is becoming a model system for experimental microbial community analyses as well [17].

***Methods**: D. melanogaster* (strain: w^{1118}) were sampled in different ways. Five samples of 5 and 10 female flies each were dissected as previously described and fecal spots of five independent culture vials were sampled and extracted with the MOBIO Soil Kit [18] to contrast the tissue associated and fecal microbial communities of *D. melanogaster*. No enrichment for bacterial DNA was used.

***Results**:* Among *D. melanogaster* samples we observe a lower alpha diversity in the shotgun profiles compared to amplicon based estimates in fecal and gut tissue samples (Figure S9, Figure S10, left panels). Community complexity, as measured by Shannon H index, is slightly higher for V1V2-two step compared to the other methods for amplicon generation. Genus richness however is highest in V3V4- one step derived samples. Analysis of beta diversities shows only little differentiation between 16S rRNA gene amplicon- and shotgun-derived profiles based on differences in abundance and more pronounced when only the presence of genera is considered (Figure S9, Figure S10, middle panels; Table S4). However, community variability is only slightly influenced by sequencing technique and shows a higher community variability of bacterial abundances in gut samples analyzed via MEGAN (Figure S9, Figure S10, right panels).

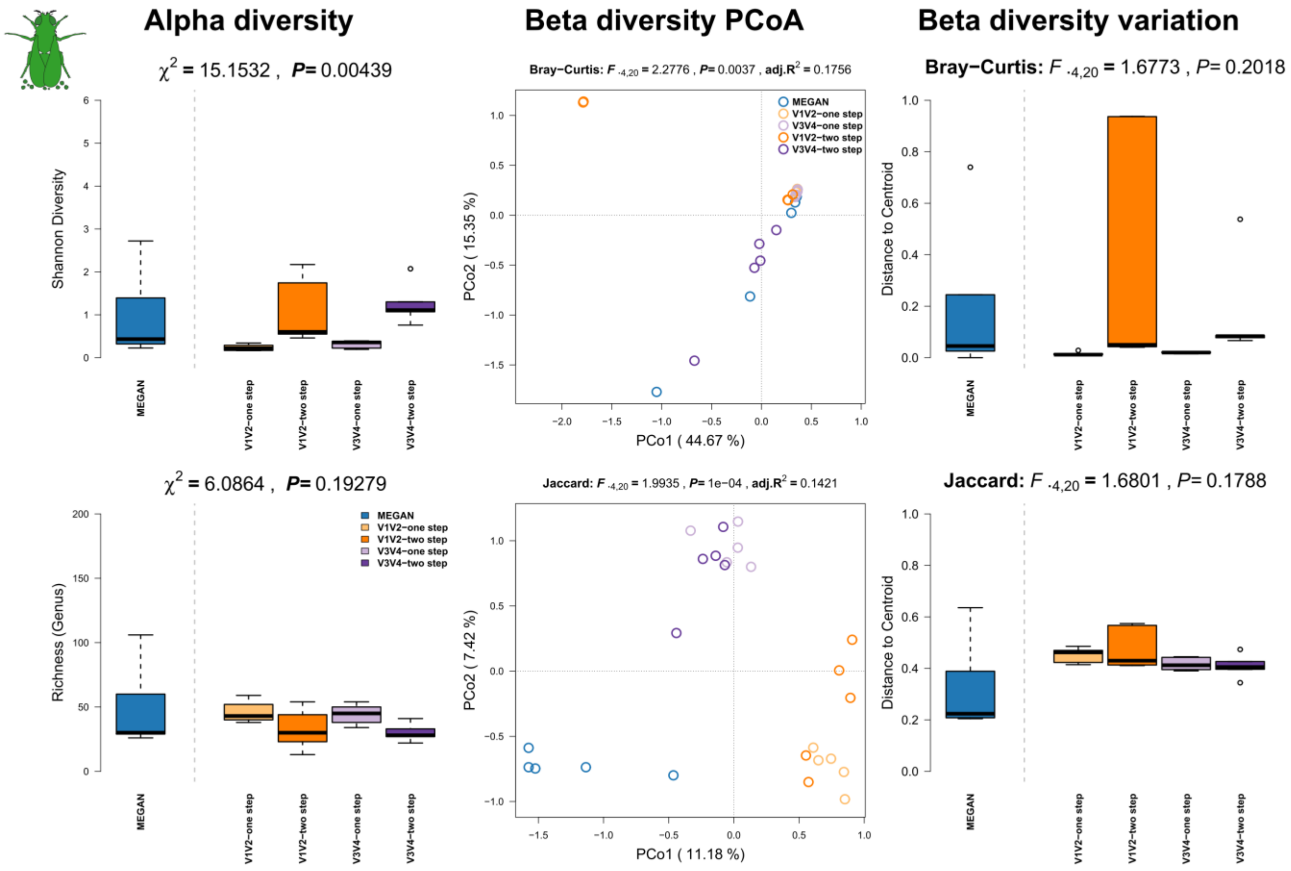

Figure S9: Comparison of community alpha diversity, composition, and variability of *Drosophila melanogaster* fecal microbial communities among 16S rRNA gene amplicon and shotgun based pipelines. Comparisons are tested via approximate Kruskal-Wallis test (alpha diversity), PERMANOVA (beta diversity), and permutative anova (variation of beta diversity). Community profiles were derived from 16S rRNA gene amplicon sequencing (V1V2, V3V4, one step, two step), MEGAN based classification (short reads). Sample sizes for the different approaches are $N_{shotgun}$=5, $N_{V1V2\text{-one step}}$=5, $N_{V1V2\text{-two step}}$=5, $N_{V3V4\text{-one step}}$=5, and $N_{V3V4\text{-two step}}$=5.

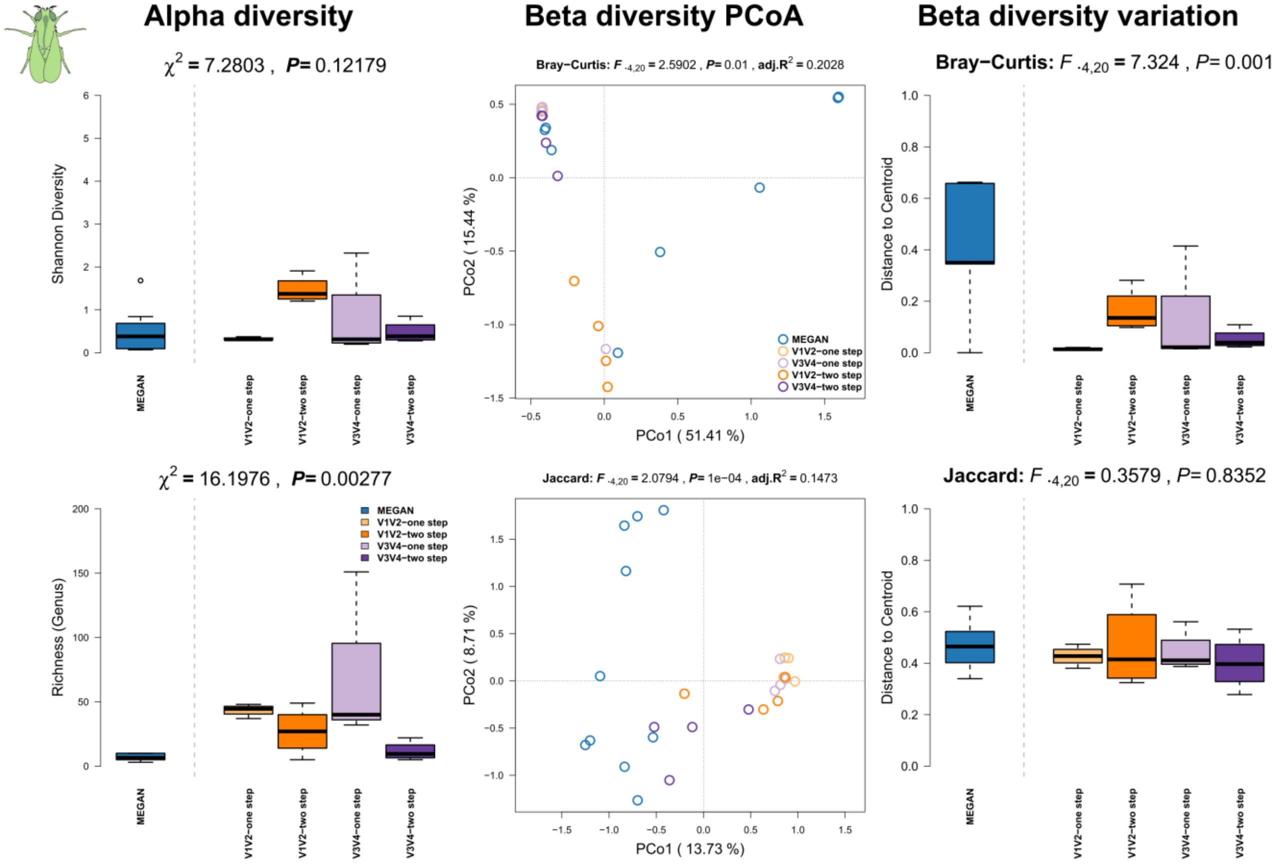

Figure S10: Comparison of community alpha diversity, composition, and variability of *Drosophila melanogaster* gut tissue associated microbial communities among 16S rRNA gene amplicon and shotgun based pipelines. Comparisons are tested via approximate Kruskal-Wallis test (alpha diversity), PERMANOVA (beta diversity), and permutative anova (variation of beta diversity). Community profiles were derived from 16S rRNA gene amplicon sequencing (V1V2, V3V4, one step, two step), MEGAN based classification (short reads). Sample sizes for the different approaches are $N_{shotgun}$=10, $N_{V1V2\text{-one step}}$=4, $N_{V1V2\text{-two step}}$=4, $N_{V3V4\text{-one step}}$=4, and $N_{V3V4\text{-two step}}$=4.

Summary *Homo sapiens* (Linnaeus, 1758):

The human (*Homo sapiens*) microbiome has been studied intensively and is presumed to be involved in wide array of diseases. Several microbiome studies have shown the influence of host genetics, environmental/life style variables, or diseases themselves [19, 20].

***Methods**:* Human feces (N=4) and biopsy samples (N=1) were sampled and extracted following the procedures as described in Wang *et al.* 2016 [19]. No enrichment for bacterial DNA was used.

***Results**:* We see again the lowest alpha diversity in the shotgun profiles using MEGAN, while all other 16S rRNA gene amplicon profiles show very similar levels of complexity and richness. Interestingly, principle coordinate analyses show no clear separation between shotgun- and

amplicon-derived profiles, except when we only consider the occurrence of genera, which separates mainly amplicon and shotgun based community profiles (Figure S11, middle panel; Table S4). There is also no clear difference in community variation noticeable among methods (Figure S11, right panel).

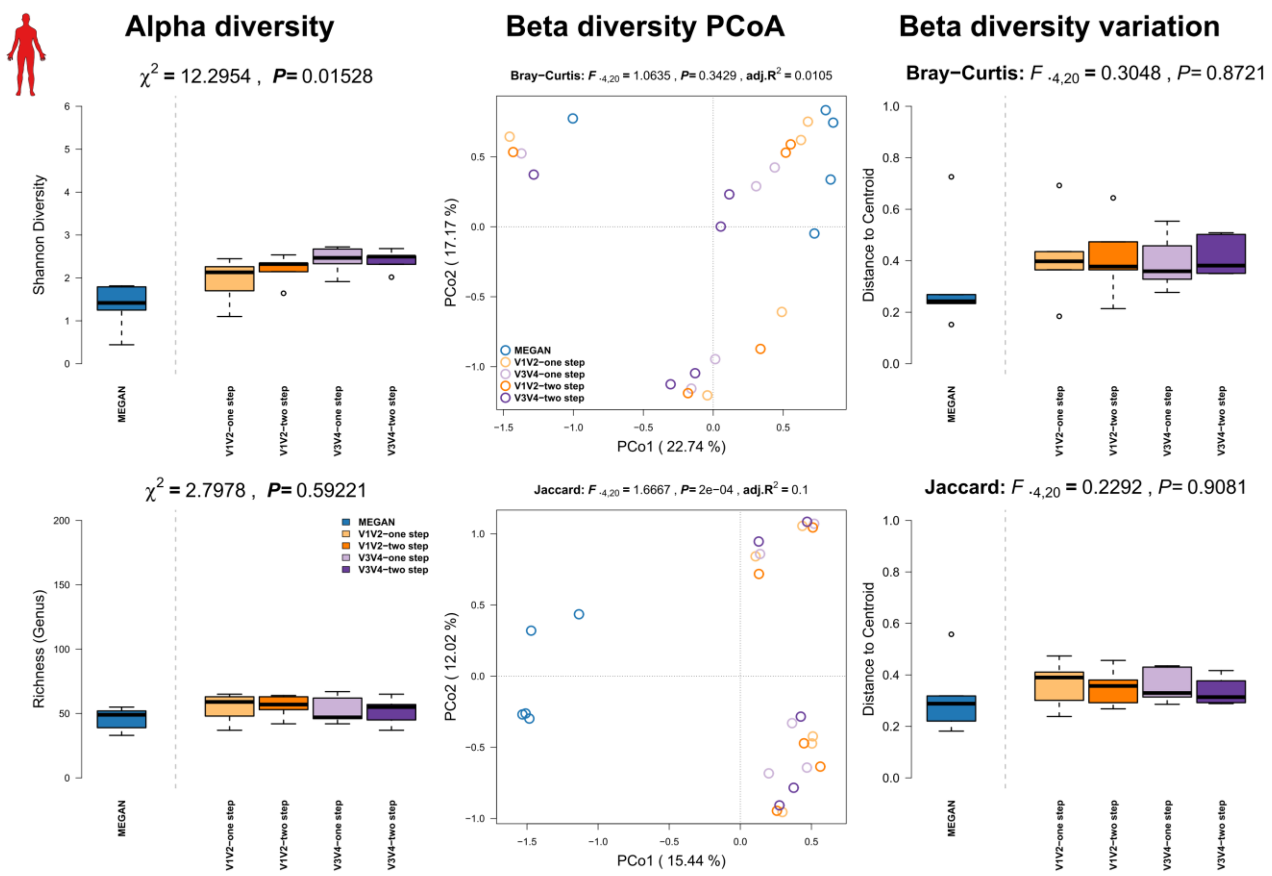

Figure S11: Comparison of community alpha diversity, composition, and variability of *Homo sapiens* associated microbial communities among 16S rRNA gene amplicon and shotgun based pipelines. Comparisons are tested via approximate Kruskal-Wallis test (alpha diversity), PERMANOVA (beta diversity), and permutative anova (variation of beta diversity). Community profiles were derived from 16S rRNA gene amplicon sequencing (V1V2, V3V4, one step, two step), MEGAN based classification (short reads). Sample sizes for the different approaches are $N_{shotgun}$=5, $N_{V1V2\text{-one step}}$=5, $N_{V1V2\text{-two step}}$=5, $N_{V3V4\text{-one step}}$=5, and $N_{V3V4\text{-two step}}$=5.

Summary *Hydra vulgaris* (Pallas, 1766)*:*

The fresh-water cnidarian *Hydra vulgaris* is an established model organism in evolutionary developmental biology belonging to the Hydrozoa. Since *Hydra* is colonized by a stable and species specific bacterial community, it is also used for the study of host-microbe interactions [21]. Its body and tissue structure resembles in some ways vertebrate intestine with the

endodermal epithelium and can be used to emulate dynamics as seen in vertebrates due to its ancestral relationship [22]. It also recently got a complete genome and has established methods for genetic manipulations [23, 24].

Methods: Two hundred adult polyps of lab raised *H. vulgaris* were used for each sample and stored at -20°C until extraction via the QiaAMP stool kit following the manufacturer's instructions. All animals were cultured under constant, identical environmental conditions including culture medium, food (first-instar larvae of *Artemia nauplii*, fed three times per week) and temperature according to standard procedures. *H. vulgaris* polyps were cultured following standard operating procedure as described in Lenhoff & Brown (1970) [25]. No enrichment for bacterial DNA was used.

Results: We see the highest alpha diversity (richness) in the shotgun profiles based on MEGAN (Figure S12, left panel), while community complexity (Shannon index) is fairly similar among sequencing techniques. The analysis of beta diversities via principle coordinate analysis shows clear separation between shotgun- and amplicon-based profiles, in particular based on differential occurrence of genera and far less based on differential abundances of taxa (Figure S12, middle panel; Table S4). V1V2- one step derived profiles are least differentiated to other methods. Variation of communities is quite comparable among the different profiling methods, however we can observe a higher variation in community membership in the 16S rRNA-two step derived community profiles (Figure S12, right panel).

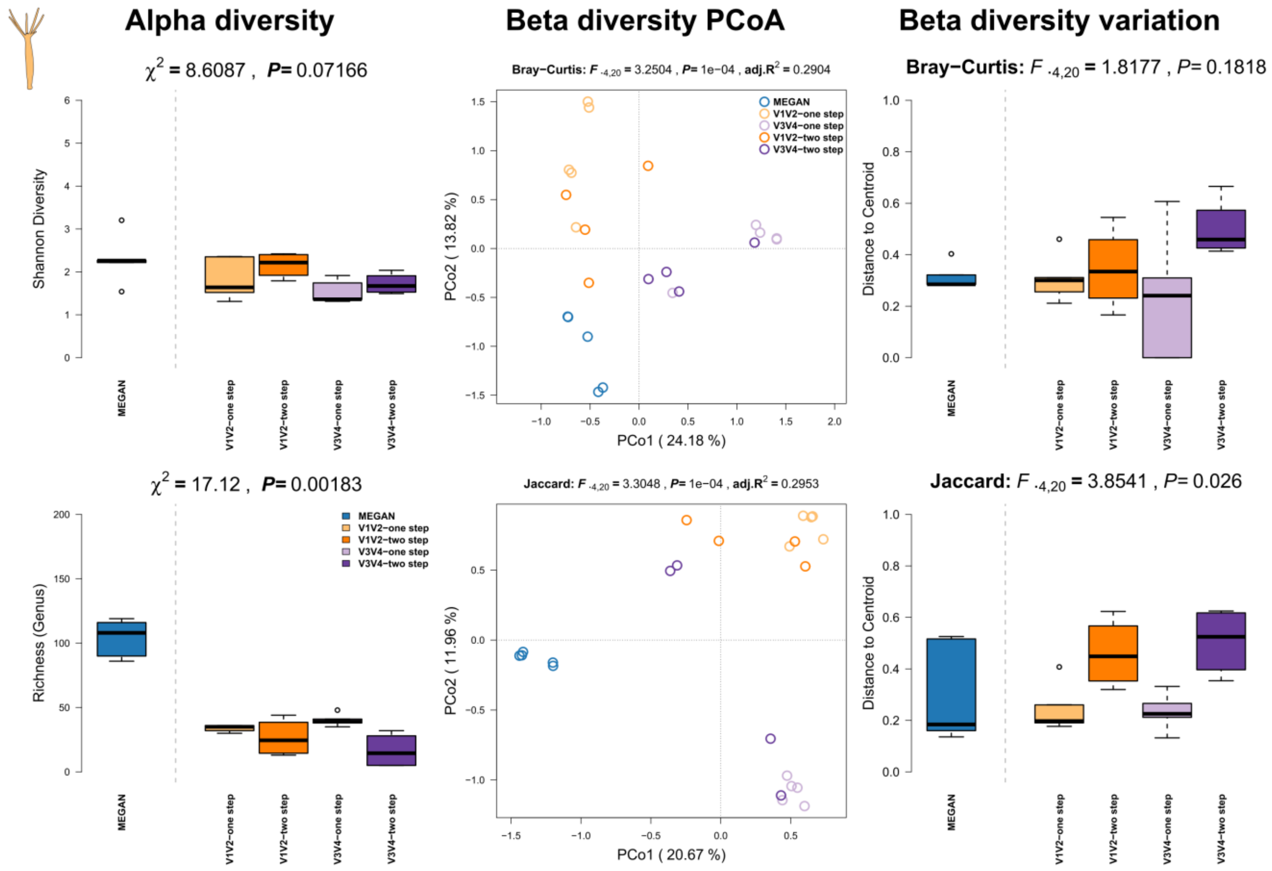

Figure S12: Comparison of community alpha diversity, composition, and variability of *Hydra vulgaris* associated microbial communities among 16S rRNA gene amplicon and shotgun based pipelines. Comparisons are tested via approximate Kruskal-Wallis test (alpha diversity), PERMANOVA (beta diversity), and permutative anova (variation of beta diversity). Community profiles were derived from 16S rRNA gene amplicon sequencing (V1V2, V3V4, one step, two step), MEGAN based classification (short reads). Sample sizes for the different approaches are $N_{shotgun}$=5, $N_{V1V2\text{-one step}}$=5, $N_{V1V2\text{-two step}}$=4, $N_{V3V4\text{-one step}}$=5, and $N_{V3V4\text{-two step}}$=4.

Summary *Mnemiopsis leidyi* (Agassiz, 1865):

Mnemiopsis leidyi is a widely distributed lobate comb jelly (Ctenophora) native to the western Atlantic coasts. *M. leidyi* has become an ecologically and economically important invasive species as it recently expanded its range to the Black, Caspian, North- and Baltic Sea through human influence. *M. leidyi* was also established as model system to study regeneration, patterning, and luminescence, as well as the evolution of the metazoans. A recently finished genome is a recent addition to the set of resources available for *M. leidyi* [26].

Methods: Individual *M. leidyi* (mean size 4 cm, N= 5) were sampled from one location in the Kiel Bight, Baltic Sea (54.330107 N, 10.149735 E) in September 2016 by using a dip net. The animals were transported immediately to the laboratory, washed thoroughly with sterile filtered

artificial seawater (ASW) to remove non-associated microbes. Separation of eukaryotic and prokaryotic cells from whole animals as well as DNA extraction were performed as described in the *A. aurita* section.

***Results**:* We can observe the highest complexity but lowest richness in the shotgun profiles using MEGAN (Figure S13, left panel). In the 16S rRNA gene amplicon profiles we see a slightly higher diversity if V3V4 amplicons are analyzed as compared to V1V2. Principle coordinate analyses show a strong separation between methods, with a strong differentiation between MEGAN based profiles and the different variable regions, particularly to V1V2 derived profiles (Figure S13, middle panel; Table S4). However, variation in community composition varied the least in the profiles based on V1V2 amplicon methods. This pattern is not observed when we only consider genus occurrences in the community profiles (Figure S13, right panel).

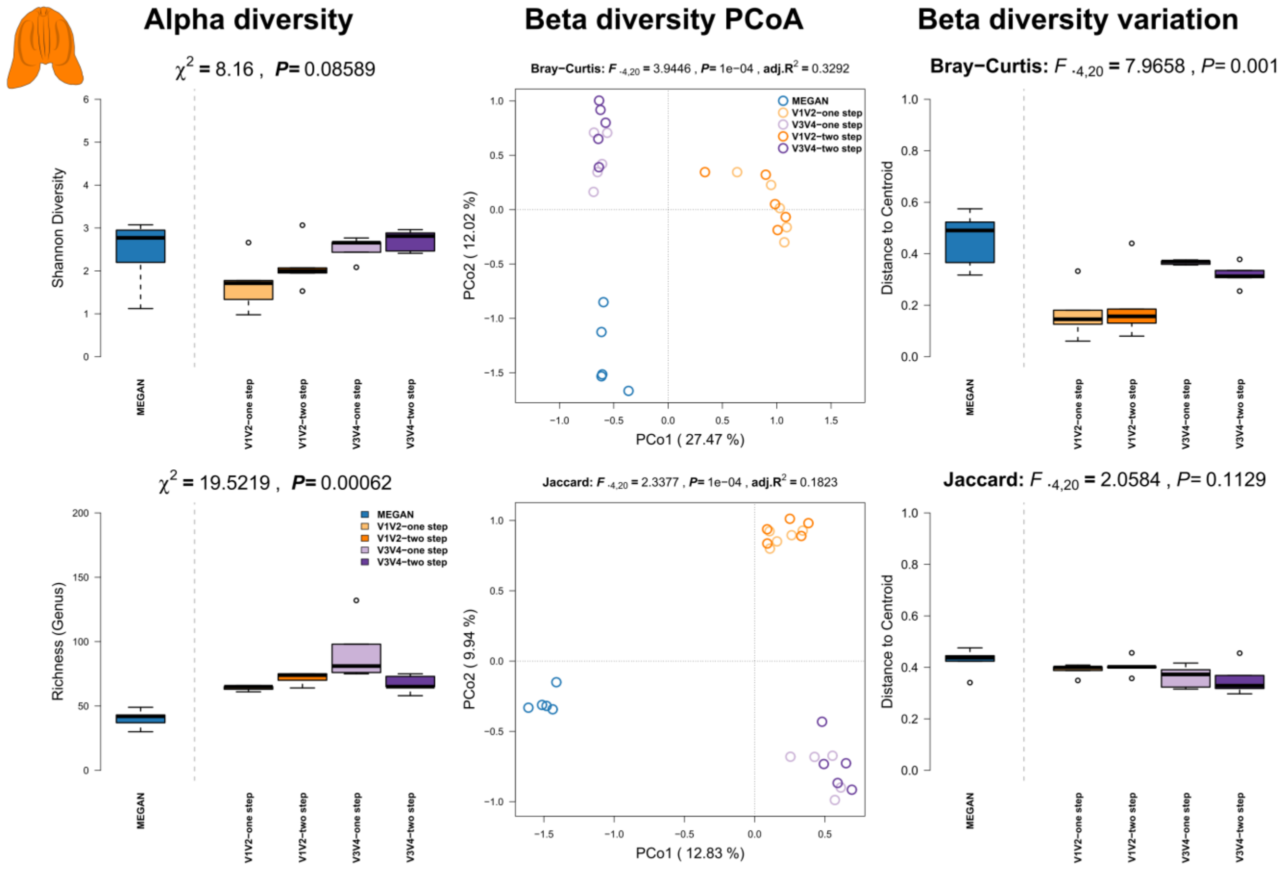

Figure S13: Comparison of community alpha diversity, composition, and variability of *Mnemiopsis leidyi* associated microbial communities among 16S rRNA gene amplicon and shotgun based pipelines. Comparisons are tested via approximate Kruskal-Wallis test (alpha diversity), PERMANOVA (beta diversity), and permutative anova (variation of beta diversity). Community profiles were derived from 16S rRNA gene amplicon sequencing (V1V2, V3V4, one step, two step), MEGAN based classification (short reads). Sample sizes for the different approaches are $N_{shotgun}$=5, $N_{V1V2\text{-one step}}$=5, $N_{V1V2\text{-two step}}$=5, $N_{V3V4\text{-one step}}$=5, and $N_{V3V4\text{-two step}}$=5.

Summary *Mus musculus* (Linnaeus, 1758):

The house mouse (*Mus musculus*) is a widely used model organism in biology and medicine. As the mouse shares many similarities to humans in terms of anatomy, physiology and genetics, it has become the preferred mammalian model for genetic research, with a wide array of genetic and physiological tools available. It has also become a widely used model for microbiome studies.

***Methods**:* Five male hybrid mice were used for sampling. The mice originate from hybrid house mouse breeding stocks of wild derived *M. m. musculus* × *M. m. domesticus* hybrids captured in 2008, kept at the Max Planck Institute in Plön (11th lab generation at time of sampling). The stocks are derived from the Bavarian hybrid zone around Munich (Germany), in particular the locations FS (N=2), HA (N=1), and TU (N=2) [27]. Handling and killing of the mice was conducted according to the German animal welfare law and Federation of European Laboratory Animal Science Associations guidelines. All mice were sacrificed with CO_2 followed by cervical dislocation. Cecal content was preserved in 1ml of RNAlater and stored at -20°C after 12h at 5°C, following centrifugation and removal of supernatant (N=5). This was later extracted using the DNA/RNA AllPrep kit (Qiagen) according to the manufacture's protocol as described previously [28, 29], with an initial bead-beating step using Lysing Matrix E tubes (MP Biomedicals). No enrichment for bacterial DNA was used.

***Results**: Mouse samples show the* the highest alpha diversity (Shannon H, richness) when community profiles are based on shotgun profiles (MEGAN) compared to the amplicon based approaches (Figure S14, left panel). We can observe further observe a clear separation between shotgun- and amplicon-derived community profiles in the principle coordinate analyses. However, we see a slightly smaller distance between shotgun- and V1V2 derived communities (Figure S14, middle panel; Table S4) and among the 16S rRNA gene amplicon profiles we observe separation between variable regions but not between amplification methods (*i.e.* V1V2 or V3V4). Furthermore, V1V2 based profiles are most variable, in particular compared to MEGAN based profiles (Figure S14, right panel).

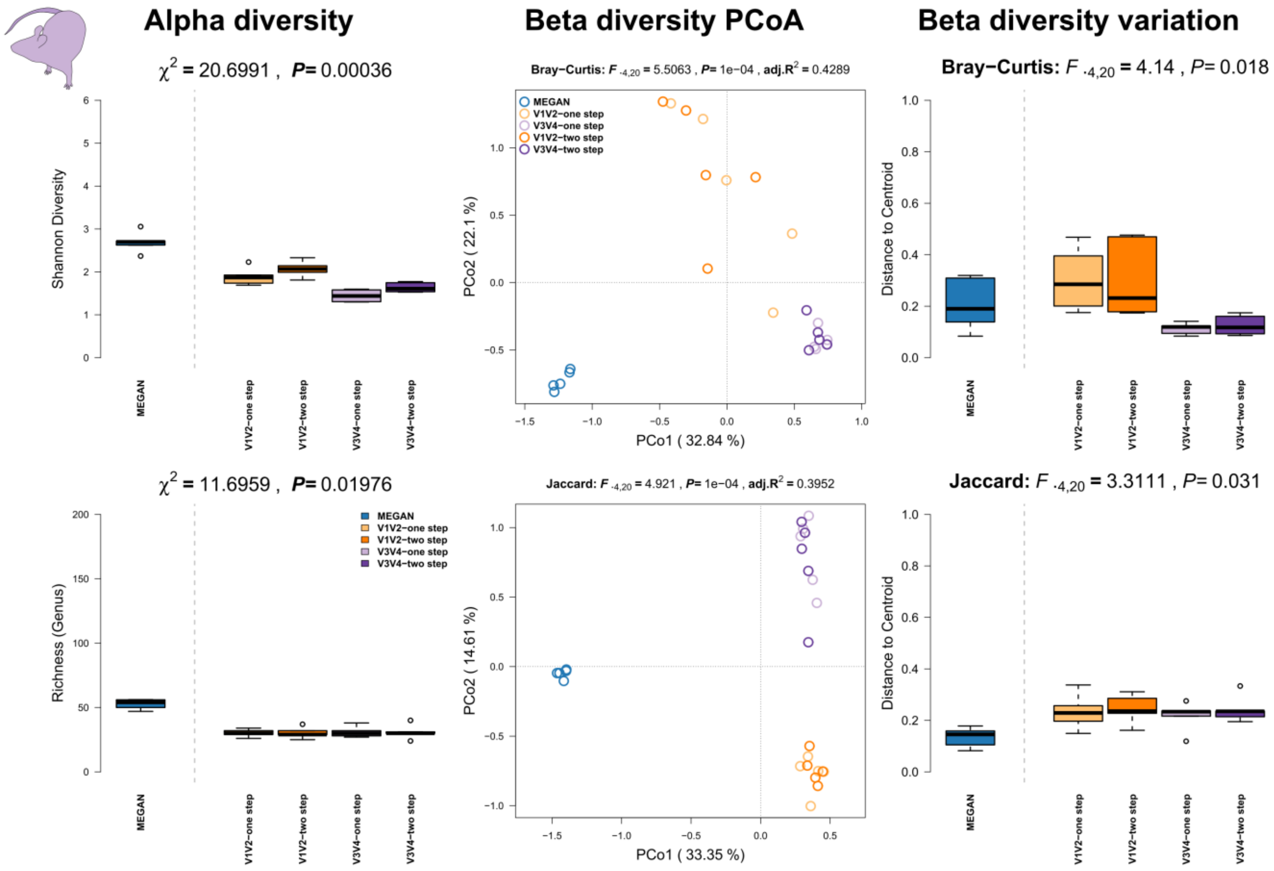

Figure S14: Comparison of community alpha diversity, composition, and variability of *Mus musculus* associated microbial communities among 16S rRNA gene amplicon and shotgun based pipelines. Comparisons are tested via approximate Kruskal-Wallis test (alpha diversity), PERMANOVA (beta diversity), and permutative anova (variation of beta diversity). Community profiles were derived from 16S rRNA gene amplicon sequencing (V1V2, V3V4, one step, two step), MEGAN based classification (short reads). Sample sizes for the different approaches are $N_{shotgun}$=5, $N_{V1V2\text{-one step}}$=5, $N_{V1V2\text{-two step}}$=5, $N_{V3V4\text{-one step}}$=5, and $N_{V3V4\text{-two step}}$=5.

Summary *Nematostella vectensis* (Stephenson, 1935):

The marine Starlet Sea Anemone *Nematostella vectensis* is a marine cnidarian (Anthozoa) living in the shallow coastal waters and marshes of Canada, the United States, and England. *N. vectensis* is a model organism for embryonic development and has an enormous adaptive potential to variable environmental factors. The animals can be cultured in the lab and reproduce sexually and asexually throughout the year.

***Methods**:* Five solitary adult lab raised polyps of *N. vectensis* were sampled and stored at -20°C until extraction via the QIAamp DNA Microbiome Kit following the manufacturer's instructions. All animals were cultured under constant, identical environmental conditions including culture medium (Artificial sea water, 16‰), food (first-instar larvae of *Artemia nauplii*, fed two times per

week) and temperature according to standard procedures (18°C, in complete darkness). No enrichment for bacterial DNA was used.

***Results**:* In contrast to the other host organisms we can see the highest alpha diversity in the one step amplicon methods, followed by MEGAN (Figure S15, left panel). In the amplicon profiles V3V4-two step method display the lowest alpha diversity. Principle coordinate analyses show differentiation between shotgun- and amplicon-sequenced samples with the lowest distance to V3V4 based community profiles (Figure S15, middle panel; Table S4). Based on the co-occurrence of genera we can observe more differentiation between amplicon- and shotgun based profiles, however V3V4 communities still cluster closest to the shotgun based communities. We can further observe a also a higher variation in the V3V4-two step PCR as well as MEGAN based community profiles (Figure S15, right panel).

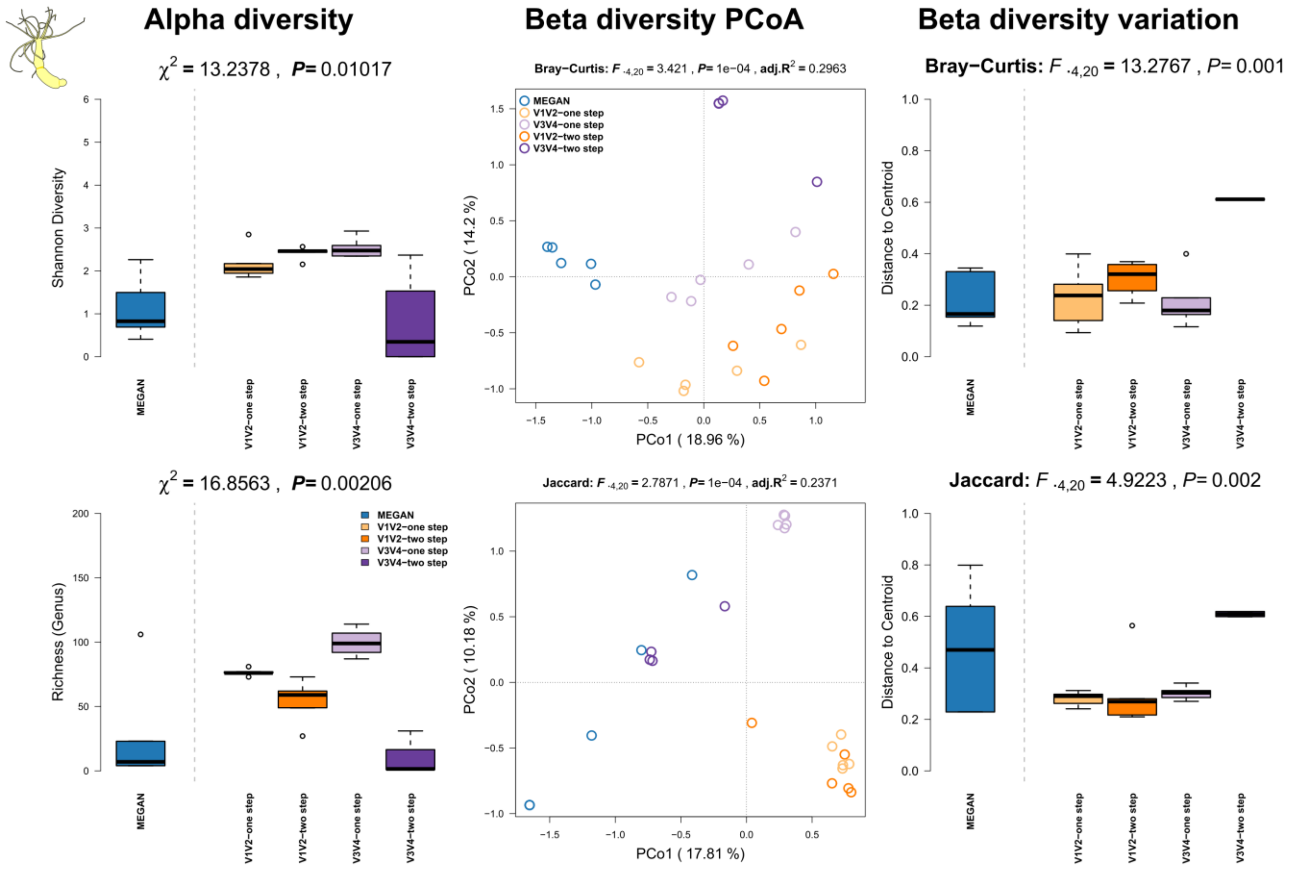

Figure S15: Comparison of community alpha diversity, composition, and variability of *Nematostella vectensis* associated microbial communities among 16S rRNA gene amplicon and shotgun based pipelines. Comparisons are tested via approximate Kruskal-Wallis test (alpha diversity), PERMANOVA (beta diversity), and permutative anova (variation of beta diversity). Community profiles were derived from 16S rRNA gene amplicon sequencing (V1V2, V3V4, one step, two step), MEGAN based classification (short reads). Sample sizes for the different approaches are $N_{shotgun}$=5, $N_{V1V2\text{-one step}}$=5, $N_{V1V2\text{-two step}}$=5, $N_{V3V4\text{-one step}}$=5, and $N_{V3V4\text{-two step}}$=4.

Summary *Triticum aestivum* (Linnaeus, 1758):

Triticum aestivum a member of the *Poaceae* is the ancestral hybrid of *Triticum dicoccum* and *Aegilops tauschii* and, potentially first cultured 7000 yr. a.d. in the fertile crescent. It is one of the most widely distributed crops, used for bread, cereal and starch. Due to its industrial use it is of high genetic homogeneity. Grains and their associated microbial communities might be beneficial and interact during germination, as well as be a direct route of heritability from a fully grown plant to the next generation via the seed [30].

***Methods**:* DNA was extracted from grains (0.1 gram) of commercially purchased *Triticum aestivum* (Davert GmbH, brand name: Bioland Davert) (N=2) and farm raised *T. aestivum* (N=3, University farm CAU Kiel, Runal cultivar). 0.1 gram of seeds was washed in 70% ethanol and surface sterilized in 3% Sodium hypochlorite for 10 minutes. Afterwards grains were washed with sterile water. Surface sterilized grains were homogenized using Precellys (SK38; according to manufacturer's protocol). Homogenate was further processed using GeneJet Plant Genomic DNA Purification Kit (ThermoFisher Scientific). Quality of extracted DNA was tested on agarose gel. Presence of the bacterial 16S rRNA gene was confirmed by PCR using bacteria specific primers with less affinity to chloroplast signatures (799F-1391R). No enrichment for bacterial DNA was used.

***Results**:* Although a high percentage of sequences has been lost due to host sequence contamination, we could still perform the majority of analyses. The highest alpha diversity has been recovered in the 16S rRNA gene amplicon based samples, particularly in the V1V2-two step (also highest richness), while the shotgun based community profiles show the lowest diversity (Figure S16, left panel). Principle coordinate analyses show a clear separation between shotgun- and amplicon-derived profiles (Figure S16, middle panel; Table S4), but also between 16S rRNA gene amplicon profiles by variable region (*i.e.* V1V2 or V3V4), which is further reduced when we only consider the presence of taxa between samples (Jaccard). On average, the variability of communities (genus abundance or occurrence) is highest in the 16S rRNA gene amplicon derived community profiles; in particular in the one step V1V2 based samples (Figure S16, right panel).

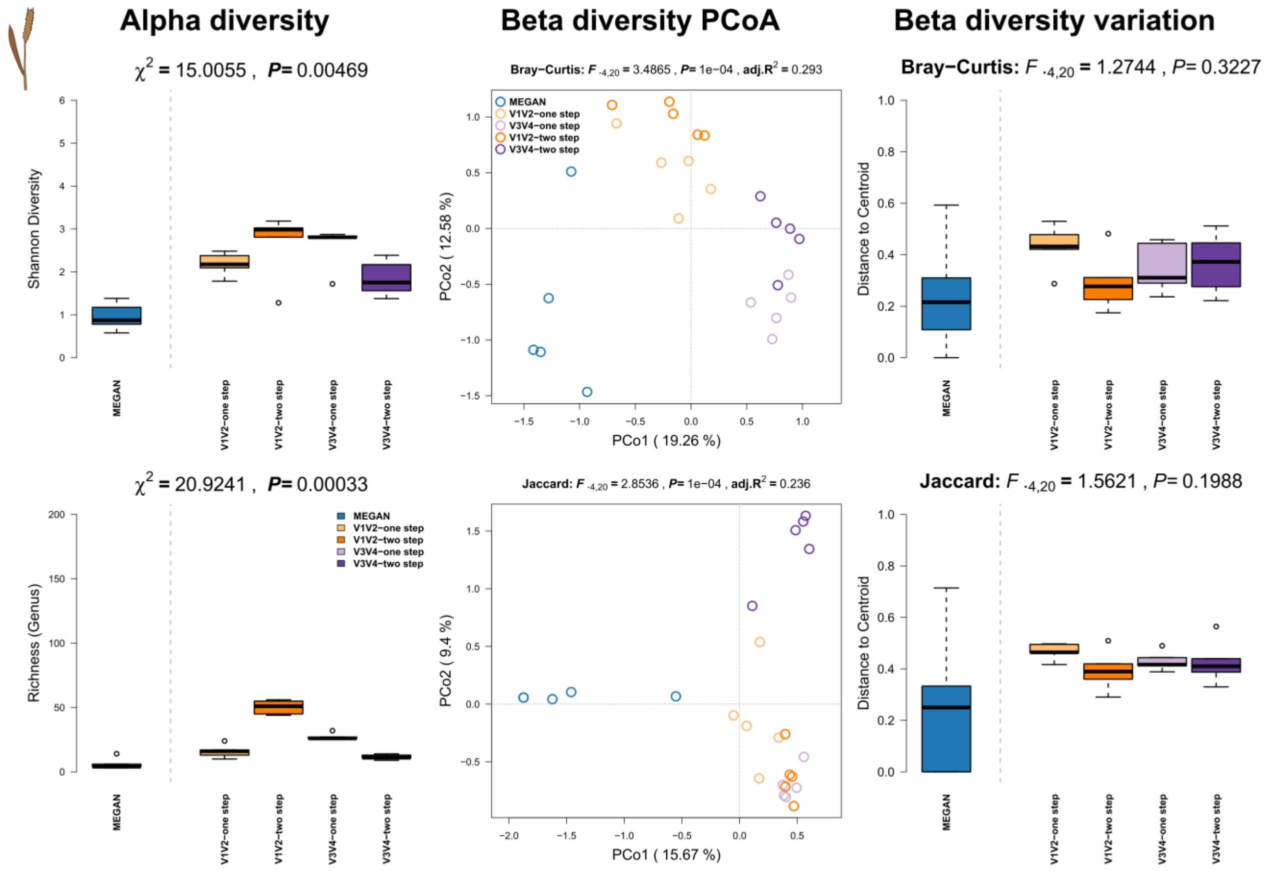

Figure S16: Comparison of community alpha diversity, composition, and variability of *Triticum aestivum* associated microbial communities among 16S rRNA gene amplicon and shotgun based pipelines. Comparisons are tested via approximate Kruskal-Wallis test (alpha diversity), PERMANOVA (beta diversity), and permutative anova (variation of beta diversity). Community profiles were derived from 16S rRNA gene amplicon sequencing (V1V2, V3V4, one step, two step), MEGAN based classification (short reads). Sample sizes for the different approaches are $N_{shotgun}$=5, $N_{V1V2\text{-one step}}$=5, $N_{V1V2\text{-two step}}$=5, $N_{V3V4\text{-one step}}$=5, and $N_{V3V4\text{-two step}}$=5.

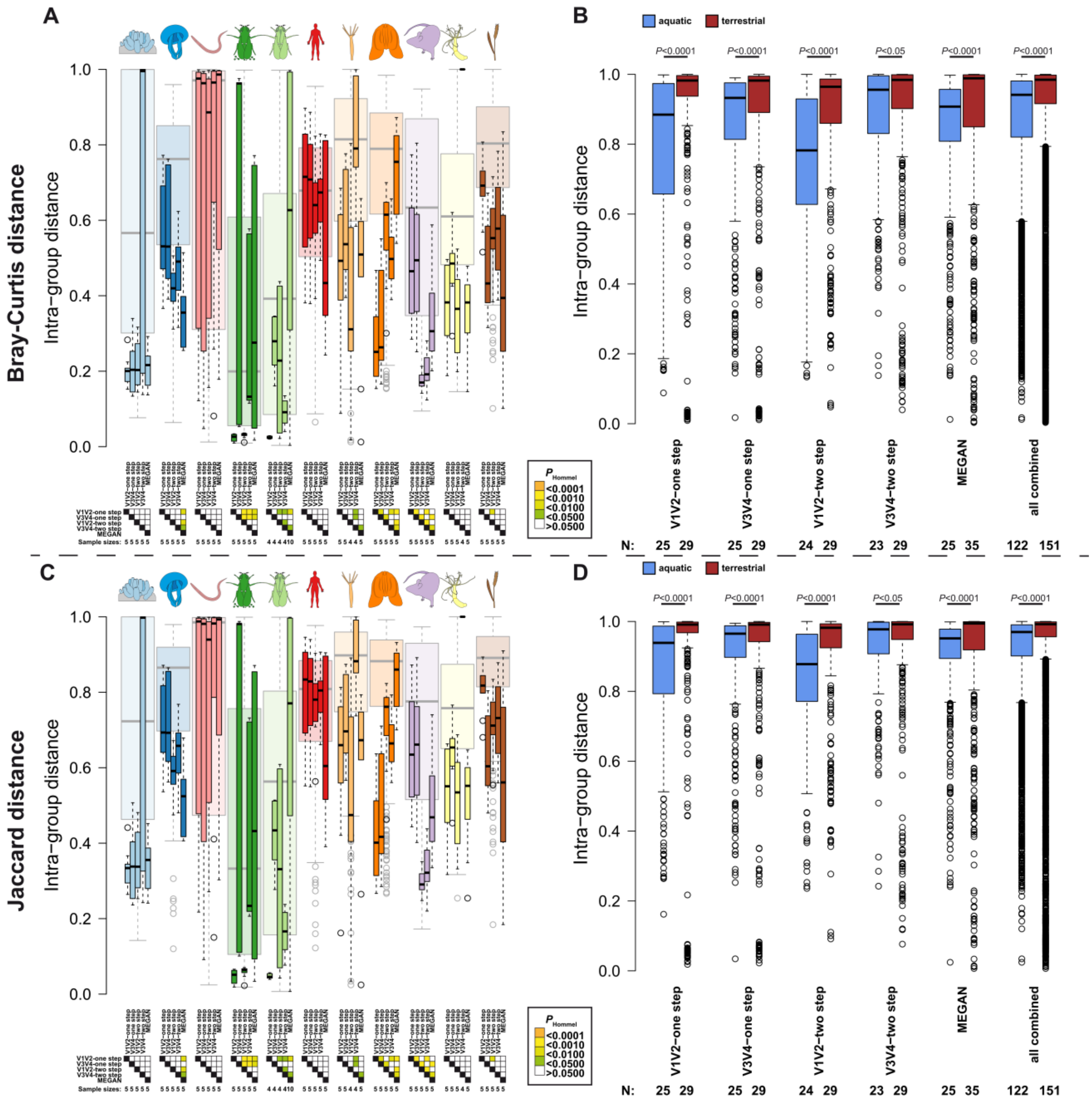

Figure S17: Boxplots visualize the pairwise distances based on shared abundance (**A, B**) and shared presence (**C, D**) between samples belonging to the respective host groups and methods (**A, C**), and host environments (**B, D**) among the different sequencing methods. Pairwise comparisons were computed via pairwise Wilcoxon tests (Hommel *P*-value adjustment) within host groups, and approximate Wilcoxon tests [6] between host environments. Significance levels after correction for multiple testing are indicated by color (for sample sizes see Table S1).

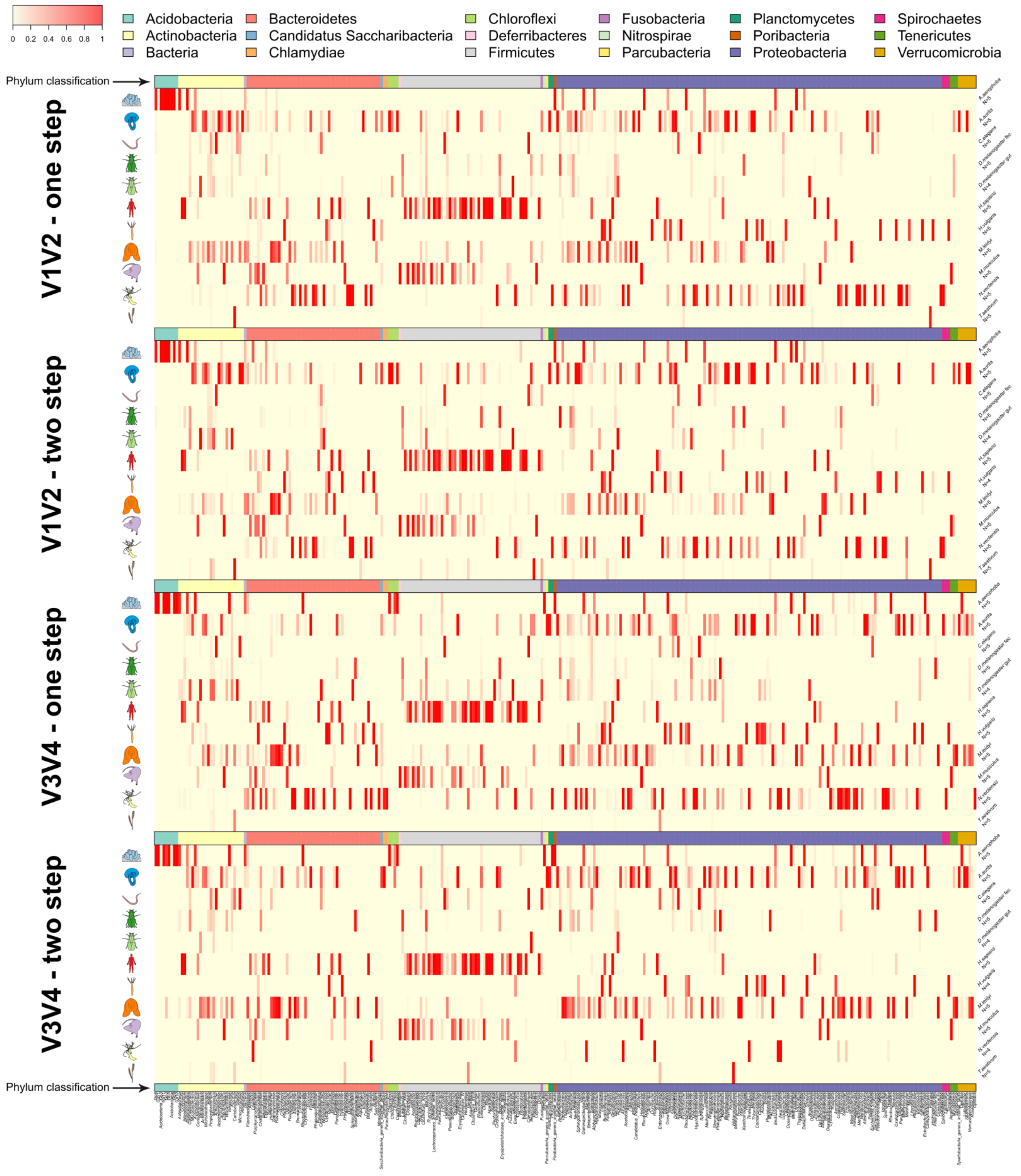

0
0.2
0.4
0.6
0.8
1
Acidobacteria
Actinobacteria
Bacteria
Bacteroidetes
Candidatus Saccharibacteria
Chlamydiae
Chloroflexi
Deferribacteres
Firmicutes
Fusobacteria
Nitrospirae
Parcubacteria
Planctomycetes
Poribacteria
Proteobacteria
Spirochaetes
Tenericutes
Verrucomicrobia
Phylum classification
V1V2 - one step
V1V2 - two step
V3V4 - one step
V3V4 - two step
Phylum classification

Figure S18: Heatmaps of indicator genera derived from the different 16S rRNA gene amplicon strategies. Heat color indicates relative abundance across sample groups and the color bar indicates phylum affiliation of the different indicators (**see Table S6**).

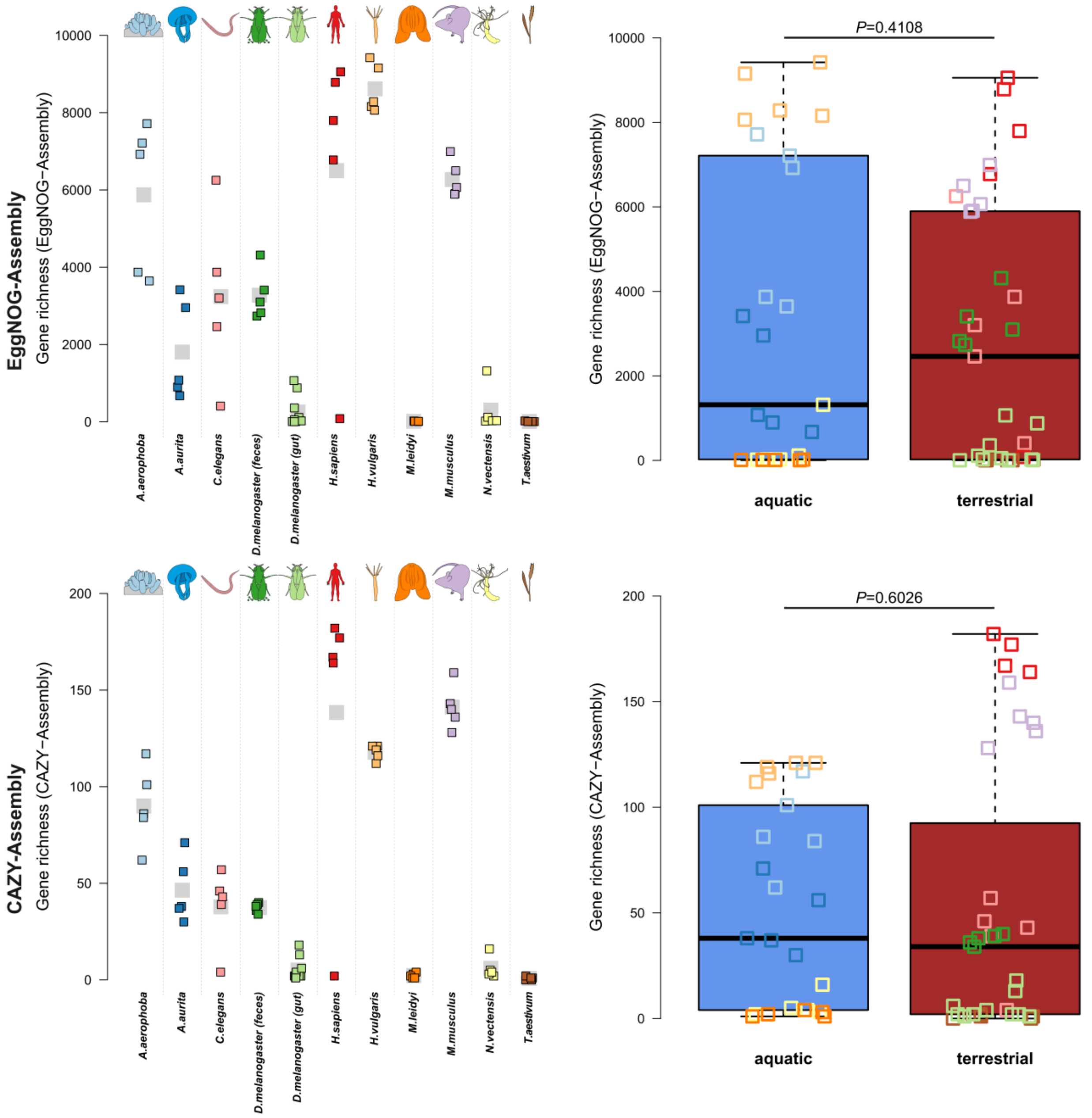

Figure S19: Functional diversity of EggNOG derived genes and CAZY predictions in the different organisms and host environments. Pairwise differences of functional diversity between hosts were tested via pairwise *t*-Tests with pooled standard variations. Approximate Wilcoxon tests were employed to compare the host environmental groups. Sample size for the host taxa is

N=5, except for D. melanogaster gut tissue (N=10). The sample size of aquatic hosts is N=25, while terrestrial hosts have a sample size of N=35 (see Table S1).

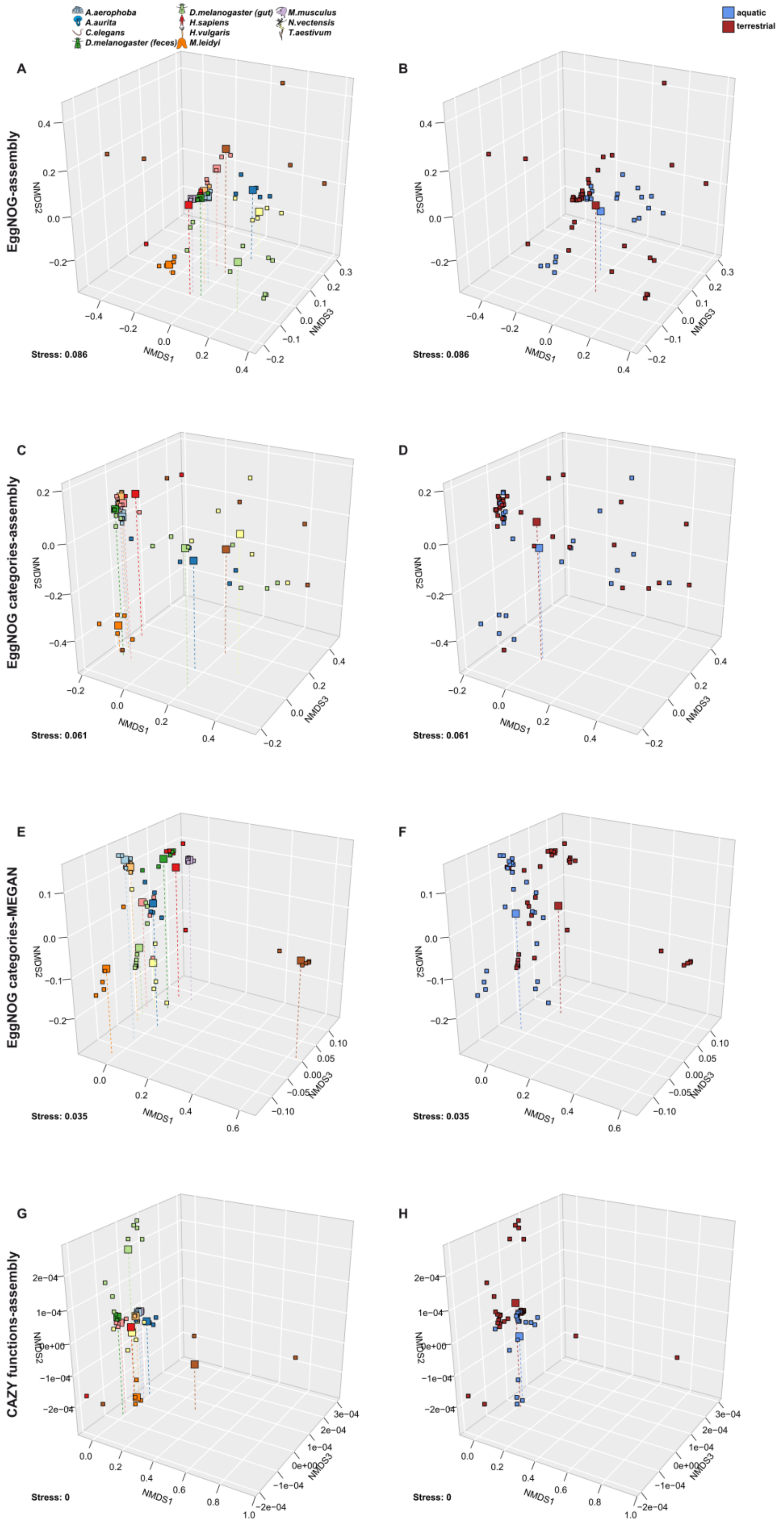

A.aerophoba
A.aurita
C.elegans
D.melanogaster (feces)
D.melanogaster (gut)
H.sapiens
H.vulgaris
M.leidyi
M.musculus
N.vectensis
T.aestivum
aquatic
terrestrial
A
B
EggNOG-assembly
NMDS1
NMDS2
NMDS3
Stress: 0.086
C
D
EggNOG categories-assembly
Stress: 0.061
E
F
EggNOG categories-MEGAN
Stress: 0.035
G
H
CAZY functions-assembly
Stress: 0

Figure S20: Non-metric Multidimensional Scaling of Bray-Curtis distances derived from abundance differences of functional components highlighting functional differences between the host organisms (**A, C, E, G; see Table 3**) and host environments (**B, D, F, H; see Table 3**). Large symbols indicate the centroid of the respective host groups and vertical lines help to determine their position in space. Functional variation of communities based on pairwise Bray-Curtis distances within host organism groups and environmental groups. Sample size for the host taxa is N=5, except for *D. melanogaster* gut tissue (N=10). The sample size of aquatic hosts is N=25, while terrestrial hosts have a sample size of N=35 (see Table S1).

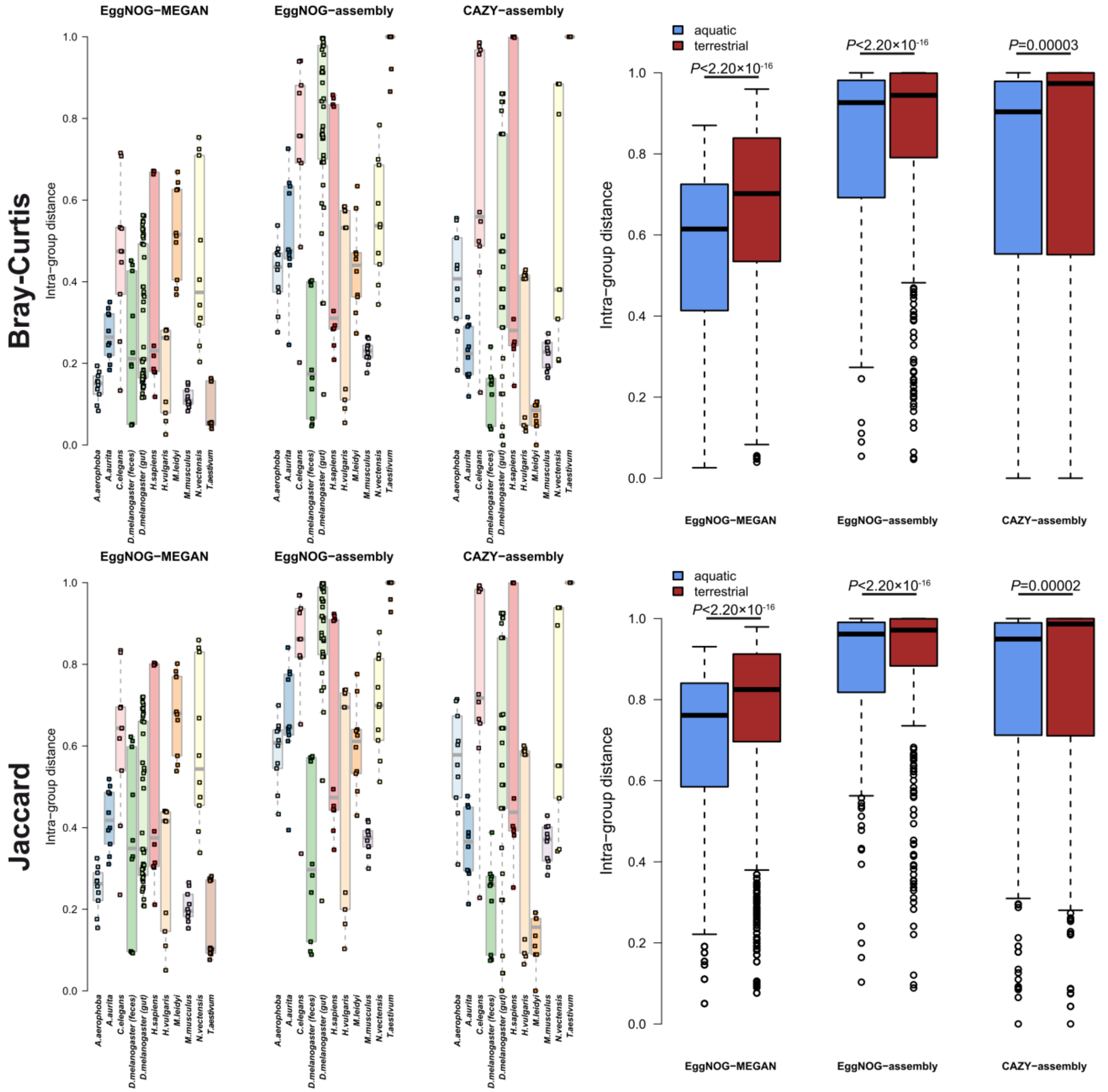

Figure S21: Functional variation of communities based on EggNOG derived genes and CAZY predictions based on pairwise Bray-Curtis distances within host organism groups and environmental groups. Terrestrial hosts show a significantly higher Functional variation than aquatic hosts. Sample size for the host taxa is N=5, except for *D. melanogaster* gut tissue (N=10). The sample size of aquatic hosts is N=25, while terrestrial hosts have a sample size of N=35 (see Table S1).

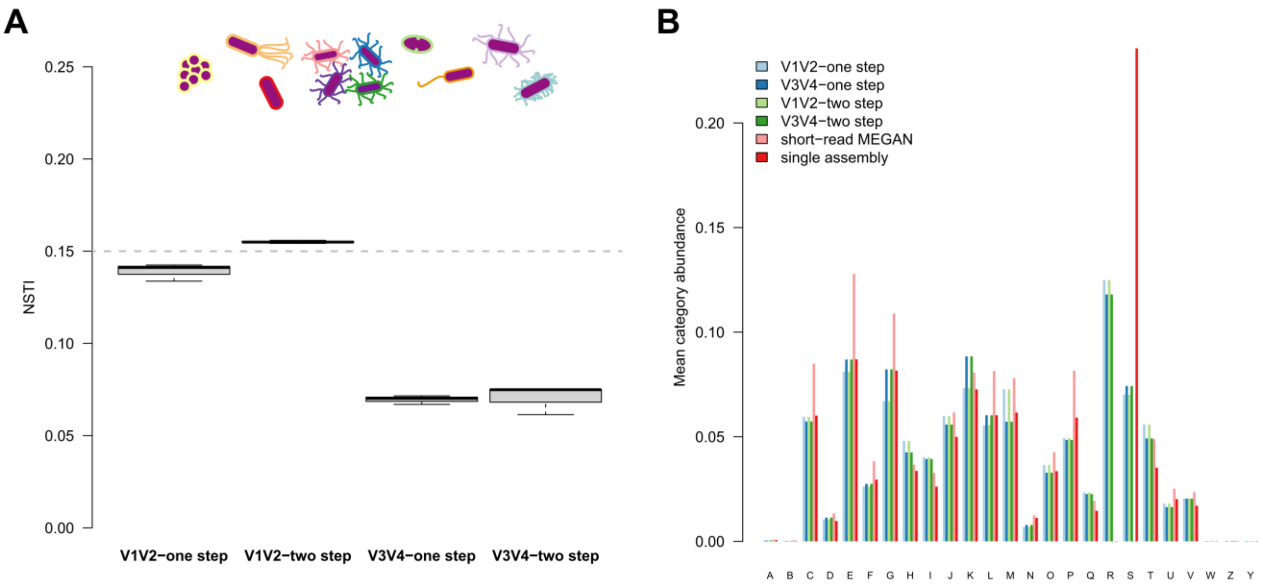

Figure S22: (A) NSTI/imputation success differences between variable regions in the mock community samples. **(B)** Barplot displays average abundances of the single functional categories derived by different 16S rRNA gene amplicon based and shotgun based techniques for mock community samples. Sample sizes for the different approaches are $N_{shotgun}$=4, $N_{V1V2\text{-}one\ step}$=3, $N_{V1V2\text{-}two\ step}$=3, $N_{V3V4\text{-}one\ step}$=3, and $N_{V3V4\text{-}two\ step}$=3.

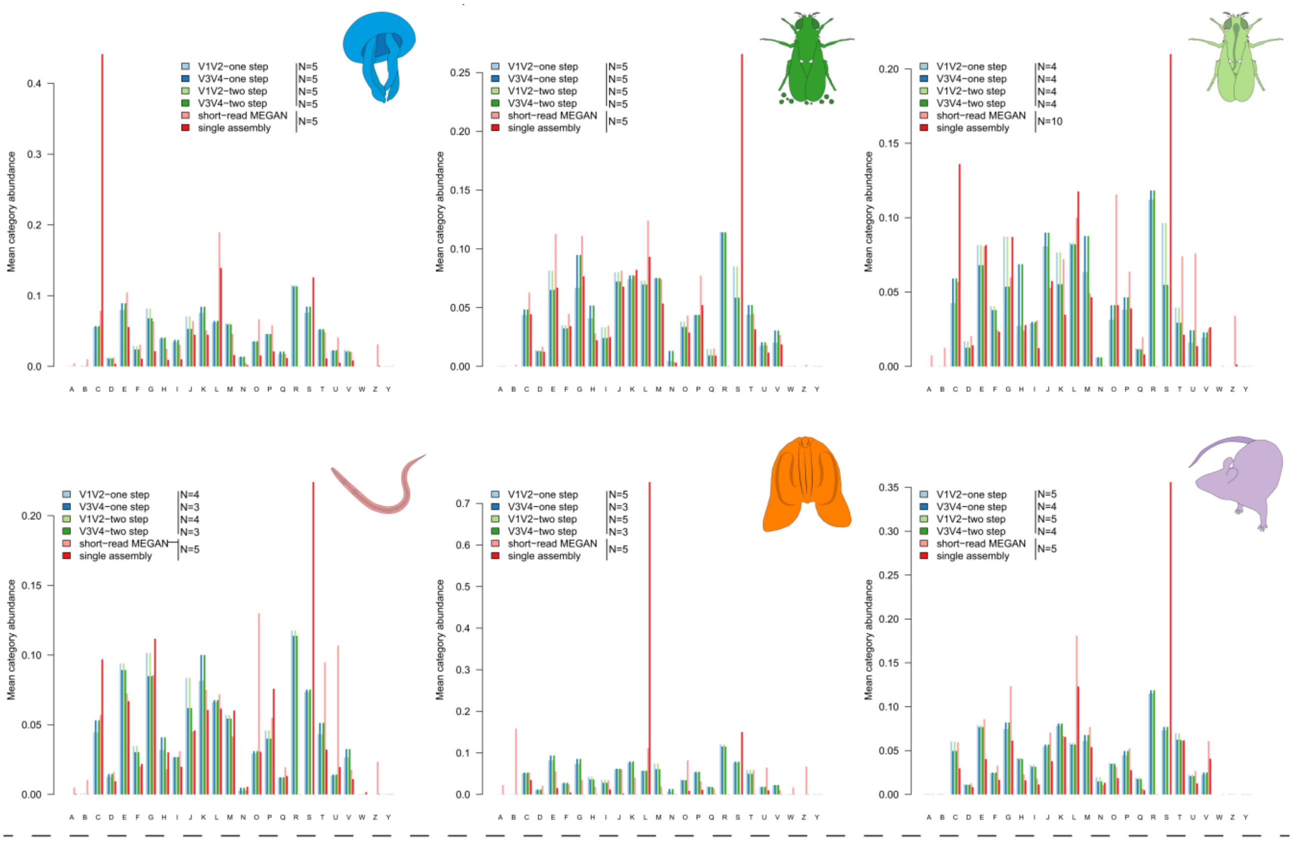

Information Storage And Processing:
J-Translation, ribosomal structure and biogenesis
A-RNA processing and modification
K-Transcription
L-Replication, recombination and repair
B-Chromatin structure and dynamics

Cellular Processes And Signaling:
D-Cell cycle control, cell division, chromosome partitioning
Y-Nuclear structure
V-Defense mechanisms
T-Signal transduction mechanisms
M-Cell wall/membrane/envelope biogenesis
N-Cell motility
Z-Cytoskeleton
W-Extracellular structures
U-Intracellular trafficking, secretion, and vesicular transport
O-Posttranslational modification, protein turnover, chaperones

Metabolism:
C-Energy production and conversion
G-Carbohydrate transport and metabolism
E-Amino acid transport and metabolism
F-Nucleotide transport and metabolism
H-Coenzyme transport and metabolism
I-Lipid transport and metabolism
P-Inorganic ion transport and metabolism
Q-Secondary metabolites biosynthesis, transport and catabolism

Poorly Characterized:
R-General function prediction only
S-Function unknown

Figure S23: Barplots display average abundances of the single functional categories derived by PICRUSt from the different 16S rRNA amplicon techniques and shotgun based annotations for specific host community samples with sufficient sample coverage.

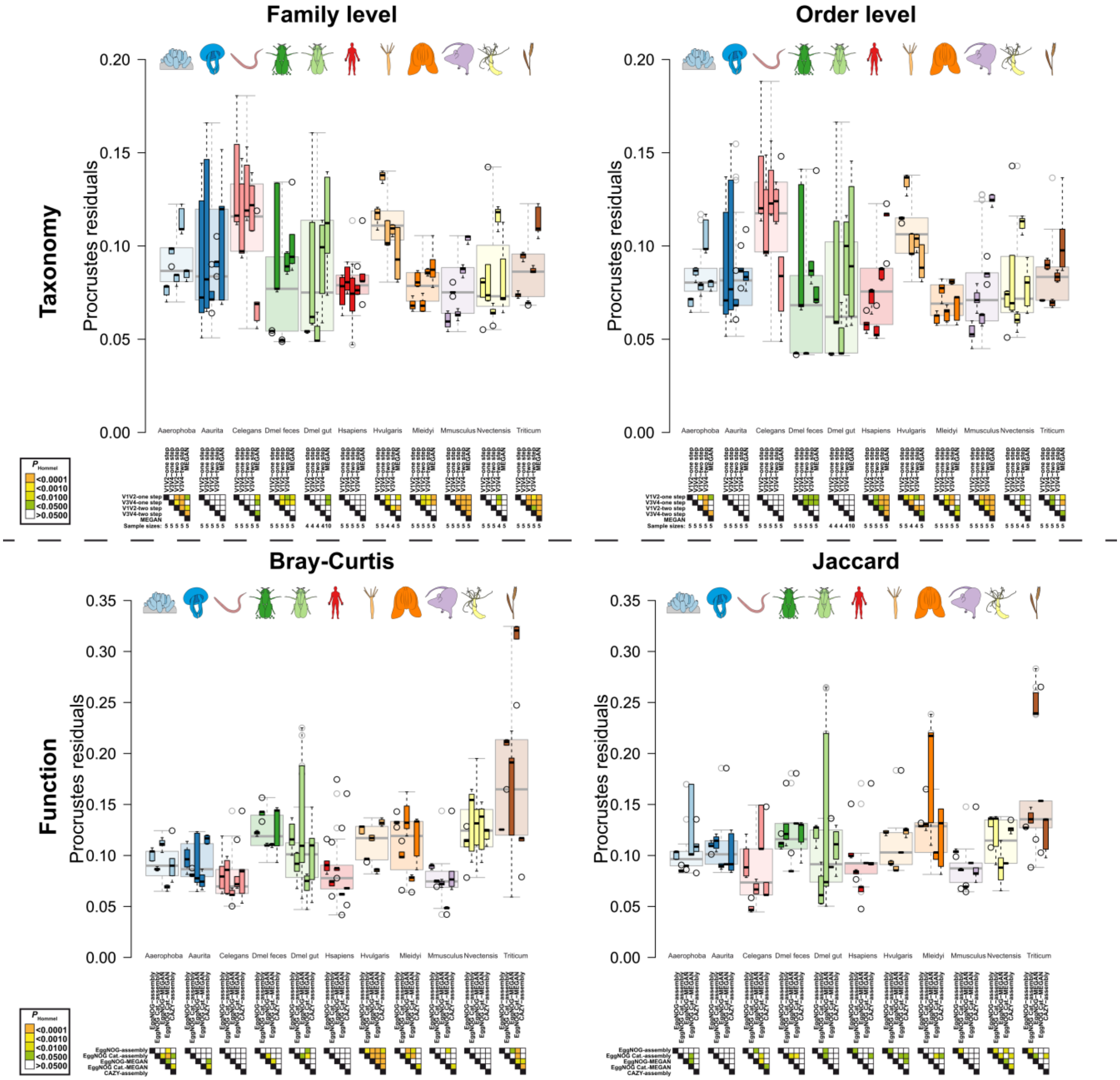

Figure S24: Upper panels visualize the residuals derived from Procrustes correlations between phylogenetic and taxonomic microbial community distances among host samples. Sample sizes are indicated below samples. Lower panels show residuals of the correlation between phylogenetic and community distances derived from functional profiles. Sample size for the host taxa is N=5, except for *D. melanogaster* gut tissue (N=10, see Table S1). Pairwise comparisons were computed via pairwise *t*-Tests (Hommel *P*-value adjustment) within host groups. Significance levels after correction for multiple testing are indicated by color.

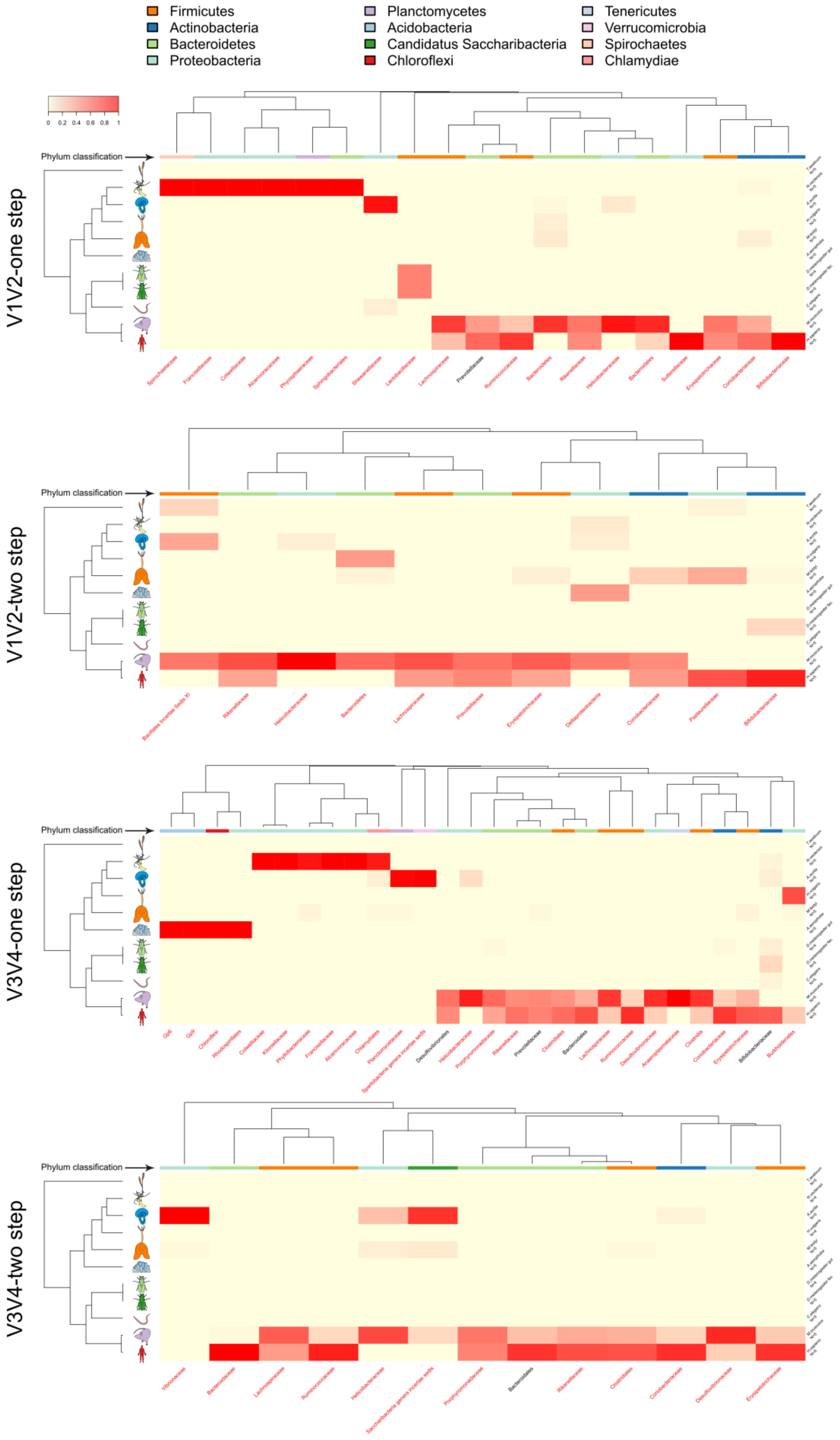

Firmicutes
Actinobacteria
Bacteroidetes
Proteobacteria
Planctomycetes
Acidobacteria
Candidatus Saccharibacteria
Chloroflexi
Tenericutes
Verrucomicrobia
Spirochaetes
Chlamydiae
0 0.2 0.4 0.6 0.8 1
Phylum classification
V1V2-one step
Spirochaetaceae
Francisellaceae
Colwelliaceae
Alcanivoracaceae
Phycisphaeraceae
Sphingobacteriales
Shewanellaceae
Lactobacillaceae
Lachnospiraceae
Prevotellaceae
Ruminococcaceae
Bacteroidetes
Rikenellaceae
Helicobacteraceae
Bacteroidales
Sutterellaceae
Erysipelotrichaceae
Coriobacteriaceae
Bifidobacteriaceae
V1V2-two step
Bacillales Incertae Sedis XI
Rikenellaceae
Helicobacteraceae
Bacteroidetes
Lachnospiraceae
Prevotellaceae
Erysipelotrichaceae
Deltaproteobacteria
Coriobacteriaceae
Pasteurellaceae
Bifidobacteriaceae
V3V4-one step
Gp6
Gp9
Chloroflexi
Rhodospirillales
Colwelliaceae
Kiloniellaceae
Phyllobacteriaceae
Francisellaceae
Alcanivoracaceae
Chlamydiales
Planctomycetaceae
Spartobacteria genera incertae sedis
Desulfovibrionales
Helicobacteraceae
Porphyromonadaceae
Rikenellaceae
Prevotellaceae
Clostridiales
Bacteroidales
Lachnospiraceae
Ruminococcaceae
Desulfovibrionaceae
Anaeroplasmataceae
Clostridia
Coriobacteriaceae
Erysipelotrichaceae
Bifidobacteriaceae
Burkholderiales
V3V4-two step
Vibrionaceae
Bacteroidaceae
Lachnospiraceae
Ruminococcaceae
Helicobacteraceae
Saccharibacteria genera incertae sedis
Porphyromonadaceae
Bacteroidales
Rikenellaceae
Clostridiales
Coriobacteriaceae
Desulfovibrionaceae
Erysipelotrichaceae

Figure S25: Heatmaps of family abundances derived from the different 16S rRNA gene amplicon strategies which associate significantly to phylogenetic distance, as determined by Moran's I test [31]. Heat colors indicate relative abundances across sample groups and the color bar indicates phylum affiliation of the different taxa (see Table S15). Row dendrogram depicts the 18S rRNA gene-based phylogeny of host species. The column dendrogram highlights the similarity of genera distributions as determined by Bray-Curtis distance and average neighbor clustering. Taxon names in red indicate a significant association as an indicator taxon (for sample sizes see Table S1).

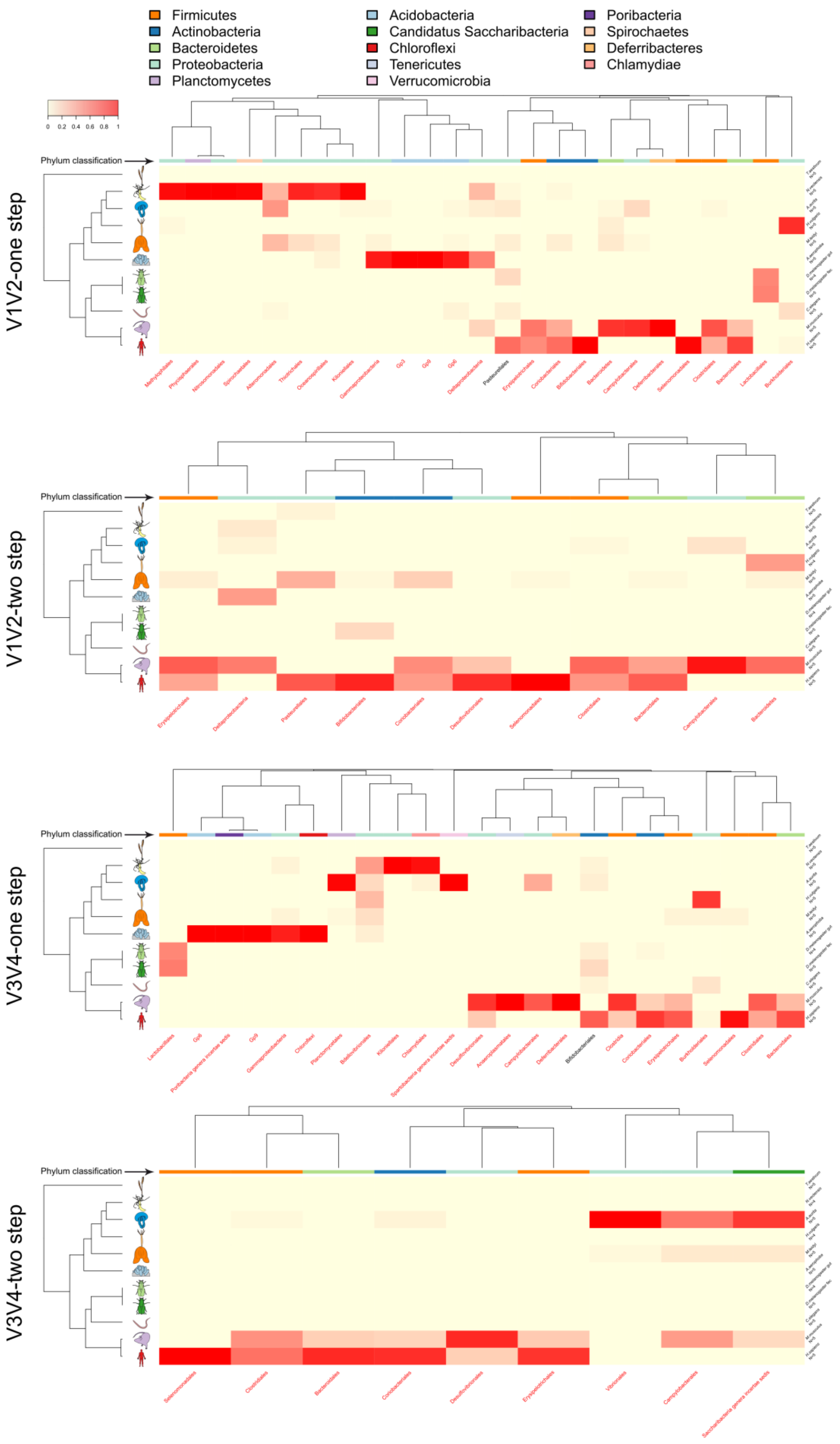

Firmicutes
Actinobacteria
Bacteroidetes
Proteobacteria
Planctomycetes
Acidobacteria
Candidatus Saccharibacteria
Chloroflexi
Tenericutes
Verrucomicrobia
Poribacteria
Spirochaetes
Deferribacteres
Chlamydiae
0 0.2 0.4 0.6 0.8 1
Phylum classification
V1V2-one step
V1V2-two step
V3V4-one step
V3V4-two step

Figure S26: Heatmaps of order abundances derived from the different 16S rRNA gene amplicon strategies which associate significantly to phylogenetic distance, as determined by Moran's I test [31]. Heat colors indicate relative abundances across sample groups and the color bar indicates phylum affiliation of the different taxa (see Table S15). Row dendrogram depicts the 18S rRNA gene-based phylogeny of host species. The column dendrogram highlights the similarity of genera distributions as determined by Bray-Curtis distance and average neighbor clustering. Taxon names in red indicate a significant association as an indicator taxon (for sample sizes see Table S1).

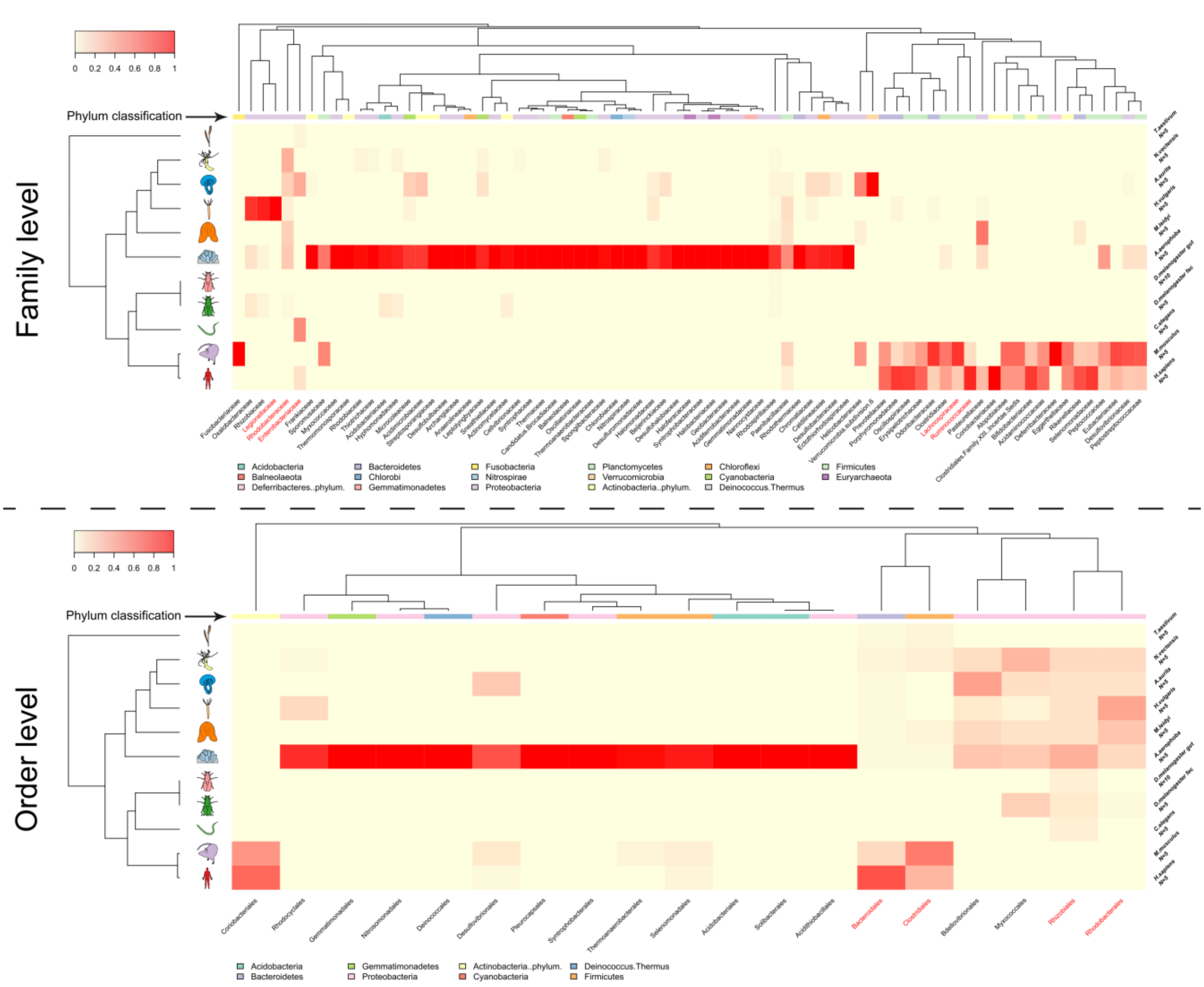

Figure S27: Heatmaps of family abundances derived from the different shotgun strategies which associate significantly to phylogenetic distance, as determined by Moran's I test [31]. Heat color

indicates relative abundance across sample groups and the color bar indicates phylum affiliation of the different indicators (see Table S16). Row dendrogram shows the 18S rRNA gene-based tree of host species (ML tree) and the column dendrogram highlights the similarity of genera distributions as determined by Bray-Curtis distance and average neighbor clustering. Taxon names in red indicate a significant association as an indicator taxon. Sample size for the host taxa is N=5, except for *D. melanogaster* gut tissue (N=10, see Table S1).

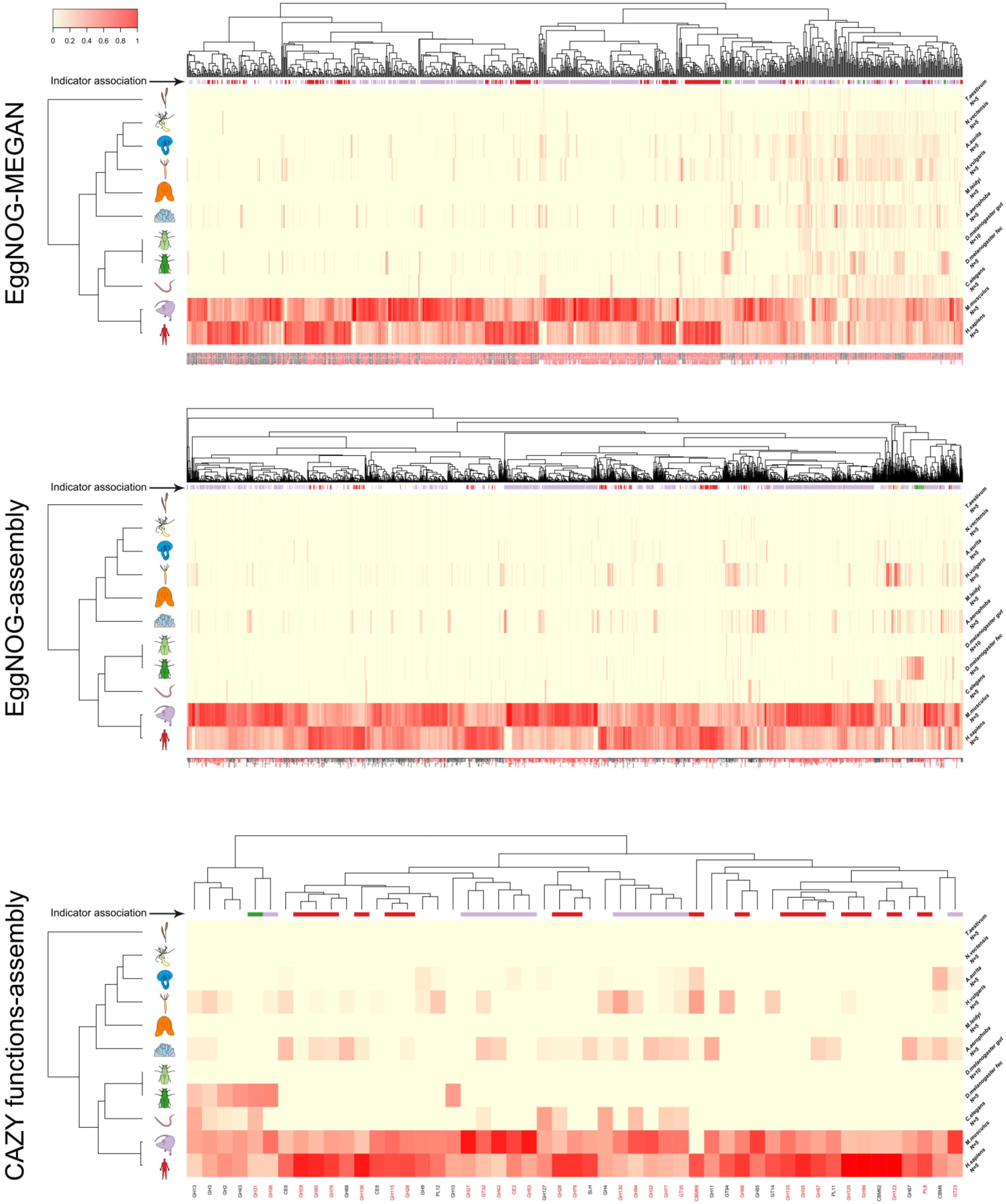

0
0.2
0.4
0.6
0.8
1
EggNOG-MEGAN
EggNOG-assembly
CAZY functions-assembly
Indicator association
T.aestivum N=5
N.vectensis N=5
A.aurita N=5
H.vulgaris N=5
M.leidyi N=5
A.aerophoba N=5
D.melanogaster gut N=10
D.melanogaster fec N=5
C.elegans N=5
M.musculus N=5
H.sapiens N=5
GH13 GH3 GH2 GH43 GH31 GH36 CE6 GH29 GH95 GH76 GH88 GH106 CE8 GH115 GH28 GH9 PL12 GH10 GH27 GT32 GH42 CE2 GH53 GH127 GH28 GH78 SLH GH4 GH130 GH94 GH32 GH77 GT35 CBM69 GH11 GT94 GH66 GH85 GT14 GH133 GH35 GH57 PL11 GH125 GH99 CBM62 GH123 GH67 PL8 CBM6 GT23

Figure S28: Heatmaps of function abundances derived from the different metagenomic functional annotation strategies associate significantly to phylogenetic distance, as determined by Moran's I test [31]. Heat colors indicate relative abundance across sample groups and the color bar indicates single host indicator associations (see Table S17-S20). Function names in red indicate a significant association as an indicator function. Row dendrogram shows the 18S rRNA gene-based tree of host species (ML tree) and the column dendrogram highlights the similarity of genera distributions as determined by Bray-Curtis distance and average neighbor clustering. Sample size for the host taxa is N=5, except for *D. melanogaster* gut tissue (N=10, see Table S1).

Supplementary Tables:

Table S1: Sample sizes of the 16S rRNA amplicon based- and shotgun based approaches for single host taxa and environments.

Table S2: Differences between expected and observed alpha diversity of the mock community, based on different shotgun- and 16S rRNA gene amplicon based community compositions (bacterial genus level), tested via a one-sample *t*-test (two-sided, *P*-values adjusted via Hommel procedure).

Table S3: Pairwise differences in mock community composition between the 16S rRNA gene amplicon and shotgun based techniques focusing on shared abundance (Bray-Curtis) and shared presence (Jaccard) of bacterial genera (PERMANOVA *P*-values based on 10'000 permutations, *P*-values adjusted via FDR procedure).

Table S4: Differences in community composition based on genus abundances and occurrences between the 16S rRNA gene amplicon and shotgun based techniques in different hosts (PERMANOVA *P*-values based on 10'000 permutations, *P*-values adjusted via Hommel procedure).

Table S5: Differences in alpha diversity estimates based on genus abundances between different hosts based on 16S rRNA amplicon and shotgun based techniques (pairwise *t*-test with Hommel adjusted *P*-values).

Table S6: Pairwise community compositional differences between the host species based on shared abundance of genera in the different amplicon techniques (Bray-Curtis). *P*-values are derived by PERMANOVA based on 10'000 permutations (*P*-values adjusted via Hommel procedure for each amplicon technique).

Table S7: Indicator genera for host taxa using different 16S rRNA gene amplicon techniques. Analyses are based on 10'000 permutations and *P*-value correction for each amplicon technique separately. Host taxa in grey highlight disagreement in their association and overlaps with shotgun techniques are listed in a separate column.

Table S8: Indicator genera for host taxa using the MEGAN based shotgun technique. Analyses are based on 10'000 permutations and *P*-value correction for each technique separately. Host taxa in grey highlight disagreement in their association overlaps with the results from the 16S rRNA gene amplicon analyses are listed in a separate column.

Table S9: Indicator genera for host environments using different 16S rRNA gene amplicon techniques. Analyses are based on 10'000 permutations and *P*-value correction for each 16S rRNA gene amplicon technique separately. Overlapping associations with the MEGAN based shotgun technique are listed in a separate column.

Table S10: Indicator genera for host environments using the different shotgun techniques. Analyses are based on 10'000 permutations and *P*-value correction for each technique separately. Overlapping associations with amplicon techniques are listed in a separate column.

Table S11: Indicator functions for host taxa using the EggNOG classification based on MEGAN. Analyses are based on 10'000 permutations with Benjamini-Yeketuli *P*-value correction.

Table S12: Indicator functions for host taxa using the EggNOG classification based on single sample assemblies and *emapper* annotation. Analyses are based on 10'000 permutations with Benjamini-Yeketuli *P*-value correction.

Table S13: Indicator functional categories for host taxa using the EggNOG classification based on single sample assemblies and *emapper* annotation and MEGAN. Analyses are based on 10'000 permutations with Benjamini-Yeketuli *P*-value correction.

Table S14: Indicator functions for host taxa using the CAZY classification based on single sample assemblies. Analyses are based on 10'000 permutations with Benjamini-Yeketuli *P*-value correction.

Table S15: Pairwise PERMANOVA comparisons between functional repertoires (EggNOG, COG) derived from single assemblies, MEGAN, and PICRUSt imputations (V1V2, V3V4, one step, two step).

Table S16: Moran's I eigenvector analyses for family and order abundances in combination with indicator analysis results for single and multiple hosts (maximum 3) using the 16S rRNA gene amplicon and metagenomic shotgun (MEGAN) based techniques (10'000 permutations). Repeated associations for single bacterial taxa, including their indicator associations are highlighted.

Table S17: Moran's I eigenvector analyses for CAZY functional abundances.

Table S18: Moran's I eigenvector analyses for EggNOG functional abundances derived from single sample assemblies.

Table S19: Moran's I eigenvector analyses for EggNOG functional abundances derived from MEGAN.

Table S20: Moran's I eigenvector analyses for EggNOG functional category abundances derived from MEGAN and single sample assemblies.

Supplementary References:

1. Huson D, Auch A, Qi J, Schuster S: **MEGAN analysis of metagenomic data**. *Genome Res* 2007, **17**(3):377 - 386.
2. Buchfink B, Xie C, Huson DH: **Fast and sensitive protein alignment using DIAMOND**. *Nat Meth* 2015, **12**(1):59-60.
3. Vacelet J: **Etude en microscopic électronique de l'association entre bacteries et spongiaires du genre Verongia (Dictyoceratida)**. *J Microsc Biol Cell* 1975, **23**:271-288.
4. Hentschel U, Hopke J, Horn M, Friedrich AB, Wagner M, Hacker J, Moore BS: **Molecular Evidence for a Uniform Microbial Community in Sponges from Different Oceans**. *Applied and Environmental Microbiology* 2002, **68**(9):4431-4440.
5. Hentschel U, Piel J, Degnan SM, Taylor MW: **Genomic insights into the marine sponge microbiome**. *Nat Rev Micro* 2012, **10**(9):641-654.
6. Hothorn T, Hornik K, Van de Wiel MA, Zeileis A: **A Lego system for conditional inference**. *American Statistician* 2006, **60**(3):257-263.
7. Legendre P, Anderson MJ: **Distance-based redundancy analysis: Testing multispecies responses in multifactorial ecological experiments**. *Ecological Monographs* 1999, **69**(1):1-24.
8. Anderson MJ: **A new method for non-parametric multivariate analysis of variance**. *Austral Ecology* 2001, **26**(1):32-46.
9. Dawson MN, Jacobs DK: **Molecular Evidence for Cryptic Species of Aurelia aurita (Cnidaria, Scyphozoa)**. *The Biological Bulletin* 2001, **200**(1):92-96.
10. Lucas CH: **Reproduction and life history strategies of the common jellyfish, Aurelia aurita, in relation to its ambient environment**. In*: 2001; Dordrecht*. Springer Netherlands: 229-246.
11. Haber M, Schungel M, Putz A, Muller S, Hasert B, Schulenburg H: **Evolutionary history of Caenorhabditis elegans inferred from microsatellites: evidence for spatial and temporal genetic differentiation and the occurrence of outbreeding**. *Mol Biol Evol* 2005, **22**(1):160-173.
12. Petersen C, Dirksen P, Prahl S, Strathmann EA, Schulenburg H: **The prevalence of Caenorhabditis elegans across 1.5 years in selected North German locations: the importance of substrate type, abiotic parameters, and Caenorhabditis competitors**. *BMC ecology* 2014, **14**:4-4.
13. Petersen C, Saebelfeld M, Barbosa C, Pees B, Hermann RJ, Schalkowski R, Strathmann EA, Dirksen P, Schulenburg H: **Ten years of life in compost: temporal and spatial variation of North German Caenorhabditis elegans populations**. *Ecology and evolution* 2015, **5**(16):3250-3263.
14. Dirksen P, Marsh SA, Braker I, Heitland N, Wagner S, Nakad R, Mader S, Petersen C, Kowallik V, Rosenstiel P *et al*: **The native microbiome of the nematode Caenorhabditis elegans: gateway to a new host-microbiome model**. *BMC Biology* 2016, **14**(1):38.
15. von der Schulenburg JH, Hancock JM, Pagnamenta A, Sloggett JJ, Majerus ME, Hurst GD: **Extreme length and length variation in the first ribosomal internal transcribed spacer of ladybird beetles (Coleoptera: Coccinellidae)**. *Mol Biol Evol* 2001, **18**(4):648-660.

16. Adams MD, Celniker SE, Holt RA, Evans CA, Gocayne JD, Amanatides PG, Scherer SE, Li PW, Hoskins RA, Galle RF *et al*: **The Genome Sequence of Drosophila melanogaster**. *Science* 2000, **287**(5461):2185-2195.
17. Fink C, Staubach F, Kuenzel S, Baines JF, Roeder T: **Noninvasive Analysis of Microbiome Dynamics in the Fruit Fly Drosophila melanogaster**. *Applied and Environmental Microbiology* 2013, **79**(22):6984-6988.
18. Fink C, von Frieling J, Knop M, Roeder T: **Drosophila Fecal Sampling**. *Bio-protocol* 2017, **7**(18):e2547.
19. Wang J, Thingholm LB, Skieceviciene J, Rausch P, Kummen M, Hov JR, Degenhardt F, Heinsen F-A, Ruhlemann MC, Szymczak S *et al*: **Genome-wide association analysis identifies variation in vitamin D receptor and other host factors influencing the gut microbiota**. *Nat Genet* 2016, **48**(11):1396-1406.
20. Rehman A, Rausch P, Wang J, Skieceviciene J, Kiudelis G, Bhagalia K, Amarapurkar D, Kupcinskas L, Schreiber S, Rosenstiel P *et al*: **Geographical patterns of the standing and active human gut microbiome in health and IBD**. *Gut* 2015, **65**(2):238-248.
21. Fraune S, Bosch TCG: **Long-term maintenance of species-specific bacterial microbiota in the basal metazoan Hydra**. *Proceedings of the National Academy of Sciences* 2007, **104**(32):13146-13151.
22. Franzenburg S, Fraune S, Künzel S, Baines JF, Domazet-Lošo T, Bosch TCG: **MyD88-deficient Hydra reveal an ancient function of TLR signaling in sensing bacterial colonizers**. *Proceedings of the National Academy of Sciences* 2012, **109**(47):19374-19379.
23. Chapman JA, Kirkness EF, Simakov O, Hampson SE, Mitros T, Weinmaier T, Rattei T, Balasubramanian PG, Borman J, Busam D *et al*: **The dynamic genome of Hydra**. *Nature* 2010, **464**:592.
24. Wittlieb J, Khalturin K, Lohmann JU, Anton-Erxleben F, Bosch TCG: **Transgenic *Hydra* allow *in vivo* tracking of individual stem cells during morphogenesis**. *Proceedings of the National Academy of Sciences* 2006, **103**(16):6208-6211.
25. Lenhoff HM, Brown RD: **Mass culture of hydra: an improved method and its application to other aquatic invertebrates**. *Laboratory Animals* 1970, **4**(1):139-154.
26. Ryan JF, Pang K, Schnitzler CE, Nguyen A-D, Moreland RT, Simmons DK, Koch BJ, Francis WR, Havlak P, Smith SA *et al*: **The genome of the ctenophore Mnemiopsis leidyi and its implications for cell type evolution**. *Science* 2013, **342**(6164):1242592.
27. Turner LM, Harr B: **Genome-wide mapping in a house mouse hybrid zone reveals hybrid sterility loci and Dobzhansky-Muller interactions**. *eLife* 2014, **3**:e02504.
28. Staubach F, Künzel S, Baines AC, Yee A, McGee BM, Bäckhed F, Baines JF, Johnsen JM: **Expression of the blood-group-related glycosyltransferase B4galnt2 influences the intestinal microbiota in mice**. *ISME J* 2012, **6**(7):1345-1355.
29. Rausch P, Basic M, Batra A, Bischoff SC, Blaut M, Clavel T, Gläsner J, Gopalakrishnan S, Grassl GA, Günther C *et al*: **Analysis of factors contributing to variation in the C57BL/6J fecal microbiota across German animal facilities**. *International Journal of Medical Microbiology* 2016, **306**(5):343-355.
30. Shade A, Jacques M-A, Barret M: **Ecological patterns of seed microbiome diversity, transmission, and assembly**. *Curr Opin Microbiol* 2017, **37**:15-22.
31. Gittleman JL, Kot M: **Adaptation: Statistics and a Null Model for Estimating Phylogenetic Effects**. *Systematic Zoology* 1990, **39**(3):227-241.
