# Supplementary material for: Comparative analysis of amplicon and metagenomic sequencing methods reveals key features in the evolution of animal metaorganisms
Source: Microbiome. 2019 Sep 14;7:133. doi: 10.1186/s40168-019-0743-1 (PMC6744666; doi:10.1186/s40168-019-0743-1)
Supplement: Supplementary file 2 — Supplementary Tables. (ZIP 1765 kb) [file 40168_2019_743_MOESM2_ESM.zip › Tab.S10.docx]

| Genus | Association | *IndVal.g* | *P* | *P*_FDR_ | Overlap with amplicon results |
| --- | --- | --- | --- | --- | --- |
| *Aeromonas* | aquatic | 0.7746 | 0.0001 | 0.0004 | V1V2-one step |
|  |  |  |  |  | V1V2-two step |
|  |  |  |  |  | V3V4-one step |
|  |  |  |  |  | V3V4-two step |
| *Lactobacillus* | terrestrial | 0.8757 | 0.0001 | 0.0004 | V1V2-one step |
|  |  |  |  |  | V1V2-two step |
|  |  |  |  |  | V3V4-one step |
|  |  |  |  |  | V3V4-two step |
| *Paracoccus* | aquatic | 0.7869 | 0.0005 | 0.0014 |  |
| *Vibrio* | aquatic | 0.9164 | 0.0001 | 0.0004 | V1V2-one step |
|  |  |  |  |  | V1V2-two step |
|  |  |  |  |  | V3V4-one step |
|  |  |  |  |  | V3V4-two step |
