# Supplementary material for: Comparative analysis of amplicon and metagenomic sequencing methods reveals key features in the evolution of animal metaorganisms
Source: Microbiome. 2019 Sep 14;7:133. doi: 10.1186/s40168-019-0743-1 (PMC6744666; doi:10.1186/s40168-019-0743-1)
Supplement: Supplementary file 2 — Supplementary Tables. (ZIP 1765 kb) [file 40168_2019_743_MOESM2_ESM.zip › Tab.S2.docx]

| Sequencing |  | Shannon |  | Richness |  |
| --- | --- | --- | --- | --- | --- |
| technique | Data | *P* | *P*_Hommel_ | *P* | *P*_Hommel_ |
| shotgun | MEGAN | 0.00005 | 0.00025 | 0.00009 | 0.00036 |
| amplicon | V1V2-one step | 0.71781 | 0.71781 | 0.00593 | 0.01186 |
|  | V3V4-one step | 0.00476 | 0.01904 | 0.01343 | 0.01343 |
|  | V1V2-two step | 0.06316 | 0.18948 | 0.00001 | 0.00005 |
|  | V3V4-two step | 0.24052 | 0.48104 | 0.00765 | 0.01343 |
