# Supplementary material for: Comparative analysis of amplicon and metagenomic sequencing methods reveals key features in the evolution of animal metaorganisms
Source: Microbiome. 2019 Sep 14;7:133. doi: 10.1186/s40168-019-0743-1 (PMC6744666; doi:10.1186/s40168-019-0743-1)
Supplement: Supplementary file 2 — Supplementary Tables. (ZIP 1765 kb) [file 40168_2019_743_MOESM2_ESM.zip › Tab.S3.docx]

| Distance | Data 1 | Data 2 | *DF* | *F* | *P* | *adj. P* (FDR) | *R*^2^ | *adj.* *R*^2^ |
| --- | --- | --- | --- | --- | --- | --- | --- | --- |
| Bray-Curtis | MEGAN | V1V2-one step | 1,5 | 17,66918 | 0,02857 | 0,07143 | 0,77944 | 0,73532 |
|  | MEGAN | V1V2-two step | 1,5 | 26,13468 | 0,02857 | 0,07143 | 0,83941 | 0,80729 |
|  | MEGAN | V3V4-one step | 1,5 | 34,87182 | 0,02857 | 0,07143 | 0,87460 | 0,84952 |
|  | MEGAN | V3V4-two step | 1,5 | 20,11927 | 0,02857 | 0,07143 | 0,80095 | 0,76114 |
|  | V1V2-one step | V1V2-two step | 1,4 | 2,95578 | 0,10000 | 0,10000 | 0,42494 | 0,28117 |
|  | V1V2-one step | V3V4-one step | 1,4 | 5,05518 | 0,10000 | 0,10000 | 0,55826 | 0,44783 |
|  | V1V2-one step | V3V4-two step | 1,4 | 3,68343 | 0,10000 | 0,10000 | 0,47940 | 0,34925 |
|  | V1V2-two step | V3V4-one step | 1,4 | 10,32445 | 0,10000 | 0,10000 | 0,72076 | 0,65095 |
|  | V1V2-two step | V3V4-two step | 1,4 | 3,77009 | 0,10000 | 0,10000 | 0,48521 | 0,35651 |
|  | V3V4-one step | V3V4-two step | 1,4 | 4,04128 | 0,10000 | 0,10000 | 0,50257 | 0,37821 |
| Jaccard | MEGAN | V1V2-one step | 1,5 | 9,21890 | 0,02857 | 0,07143 | 0,64836 | 0,57803 |
|  | MEGAN | V1V2-two step | 1,5 | 10,45100 | 0,02857 | 0,07143 | 0,67640 | 0,61168 |
|  | MEGAN | V3V4-one step | 1,5 | 7,57062 | 0,02857 | 0,07143 | 0,60225 | 0,52270 |
|  | MEGAN | V3V4-two step | 1,5 | 11,17236 | 0,02857 | 0,07143 | 0,69083 | 0,62900 |
|  | V1V2-one step | V1V2-two step | 1,4 | 1,12051 | 0,30000 | 0,30000 | 0,21883 | 0,02353 |
|  | V1V2-one step | V3V4-one step | 1,4 | 1,86844 | 0,10000 | 0,12500 | 0,31839 | 0,14798 |
|  | V1V2-one step | V3V4-two step | 1,4 | 2,38902 | 0,10000 | 0,12500 | 0,37393 | 0,21741 |
|  | V1V2-two step | V3V4-one step | 1,4 | 1,76332 | 0,10000 | 0,12500 | 0,30596 | 0,13244 |
|  | V1V2-two step | V3V4-two step | 1,4 | 2,16436 | 0,10000 | 0,12500 | 0,35111 | 0,18889 |
|  | V3V4-one step | V3V4-two step | 1,4 | 1,31324 | 0,30000 | 0,30000 | 0,24716 | 0,05895 |
