# Supplementary material for: Comparative analysis of amplicon and metagenomic sequencing methods reveals key features in the evolution of animal metaorganisms
Source: Microbiome. 2019 Sep 14;7:133. doi: 10.1186/s40168-019-0743-1 (PMC6744666; doi:10.1186/s40168-019-0743-1)
Supplement: Supplementary file 2 — Supplementary Tables. (ZIP 1765 kb) [file 40168_2019_743_MOESM2_ESM.zip › Tab.S8.docx]

| Genus | Association | IndVal.g | *P* | *P*_FDR_ | Overlap amplicon results |
| --- | --- | --- | --- | --- | --- |
| *Acetobacter* | *D.melanogaster (gut)* | 0.7060 | 0.0041 | 0.0050 | V1V2-one step |
|  |  |  |  |  | V1V2-two step |
|  |  |  |  |  | V3V4-one step |
|  |  |  |  |  | V3V4-two step |
| *Aeromonas* | *H.vulgaris* | 0.9850 | 0.0001 | 0.0002 | V1V2-one step |
|  |  |  |  |  | V1V2-two step |
|  |  |  |  |  | V3V4-one step |
| *Bacteroides* | *H.sapiens* | 0.8673 | 0.0001 | 0.0002 | V1V2-one step |
|  |  |  |  |  | V1V2-two step |
|  |  |  |  |  | V3V4-one step |
|  |  |  |  |  | V3V4-two step |
| *Escherichia* | *C.elegans* | 0.8717 | 0.0003 | 0.0004 | V1V2-one step |
|  |  |  |  |  | V1V2-two step |
| *Lactobacillus* | *D.melanogaster (feces)* | 0.7410 | 0.0001 | 0.0002 | V1V2-one step |
|  |  |  |  |  | V3V4-one step |
|  |  |  |  |  | V3V4-two step |
| *Pantoea* | *T.aestivum* | 0.9978 | 0.0001 | 0.0002 | V1V2-one step  V1V2-two step |
| *Paracoccus* | *M.leidyi* | 0.9282 | 0.0001 | 0.0002 |  |
| *Pseudomonas* | *C.elegans* | 0.8087 | 0.0109 | 0.0120 | V1V2-one step |
|  |  |  |  |  | V1V2-two step |
|  |  |  |  |  | V3V4-one step |
|  |  |  |  |  | V3V4-two step |
| *Ralstonia* | *T.aestivum* | 0.8855 | 0.0001 | 0.0002 |  |
| *Vibrio* | *N.vectensis* | 0.7760 | 0.0001 | 0.0002 | V1V2-one step |
|  |  |  |  |  | V3V4-one step |
